# Efficient generation of Knock-in/Knock-out marmoset embryo via CRISPR/Cas9 gene editing

**DOI:** 10.1038/s41598-019-49110-3

**Published:** 2019-09-03

**Authors:** Wakako Kumita, Kenya Sato, Yasuhiro Suzuki, Yoko Kurotaki, Takeshi Harada, Yang Zhou, Noriyuki Kishi, Kengo Sato, Atsu Aiba, Yasubumi Sakakibara, Guoping Feng, Hideyuki Okano, Erika Sasaki

**Affiliations:** 1 Central Institute for Experimental Animals, Kawasaki-shi, Kanagawa 210-0821 Japan; 2 Department of Molecular Biology and Biochemistry, Graduate School of Medicine, Osaka University, Suita-shi, Osaka 565-0871 Japan; 3 Laboratory of Animal Resources, Center for Disease Biology and Integrative Medicine, Graduate School of Medicine, The University of Tokyo, Hongo, Bunkyo-ku, Tokyo 113-0033 Japan; 4 McGovern Institute for Brain Research, Department of Brain and Cognitive Sciences, Massachusetts Institute of Technology, Cambridge, Massachusetts 02139 USA; 5 Laboratory for Marmoset Neural Architecture, RIKEN Center for Brain Science, Wako-shi, Saitama 351-0198 Japan; 6 Department of Biosciences and Informatics, Keio University, Yokohama-shi, Kanagawa 223-8522 Japan; 7 Department of Physiology, Keio University School of Medicine, Shinjuku-ku, Tokyo 160-8582 Japan; 8 Advanced Research Center, Keio University, Shinjuku-ku, Tokyo 160-8582 Japan

**Keywords:** Animal biotechnology, CRISPR-Cas9 genome editing

## Abstract

Genetically modified nonhuman primates (NHP) are useful models for biomedical research. Gene editing technologies have enabled production of target-gene knock-out (KO) NHP models. Target-gene-KO/knock-in (KI) efficiency of CRISPR/Cas9 has not been extensively investigated in marmosets. In this study, optimum conditions for target gene modification efficacies of CRISPR/mRNA and CRISPR/nuclease in marmoset embryos were examined. CRISPR/nuclease was more effective than CRISPR/mRNA in avoiding mosaic genetic alteration. Furthermore, optimal conditions to generate KI marmoset embryos were investigated using CRISPR/Cas9 and 2 different lengths (36 nt and 100 nt) each of a sense or anti-sense single-strand oligonucleotide (ssODN). KIs were observed when CRISPR/nuclease and 36 nt sense or anti-sense ssODNs were injected into embryos. All embryos exhibited mosaic mutations with KI and KO, or imprecise KI, of *c-kit*. Although further improvement of KI strategies is required, these results indicated that CRISPR/Cas9 may be utilized to produce KO/KI marmosets via gene editing.

## Introduction

The use of mice target gene knock-in (KI)/knock-out (KO) models is important for understanding mechanisms underlying gene function and diseases caused by genetic mutations, as well as for developing new therapies for such diseases. Specifically, a study of target-gene KO model phenotypes, resulting from the loss of gene function, may enable the functions of specific genes to be clarified. Target-gene KI models are produced by insertion of a particular gene or mutation(s) into a specific region of the target gene. They are widely used to understand the function of the regulatory region of a target gene, such as the promoter region. Generally, target-gene KI/KO animals are created via chimeric animals using the conventional method of injecting target-gene KI/KO embryonic stem cells into preimplantation embryos^1^. However, embryonic stem cells (ESCs) capable of generating chimeric animals have proven to be elusive in most species except rodents^2–7^.

More recently, several types of gene editing technologies including zinc finger nucleases (ZFNs)^8^, transcription activator-like effector nucleases (TALENs)^9^ and clustered regularly interspaced short palindromic repeat (CRISPR)/CRISPR-associated protein 9 (CRISPR/Cas9)^10,11^ have been developed. All these technologies involve artificial nucleases and induce double-strand breaks (DSB) in a specific gene of the target genome. DSB repair may lead to non-homologous end-joining causing a frameshift, which disrupts the function of the target gene. Such gene alteration techniques can be applied to preimplantation embryos, making it possible to generate target-gene KO/KI models in many animal species including NHPs that lack chimeric competent ESCs^12–14^.

The common marmoset (*Callithrix jacchus*, marmoset) is a prolific, small sized NHP that matures sexually in a relatively short period (1.5–2 years), and is quite easy to handle. Due to these biological features, the marmoset has been developed as an experimental animal. Several transgenic marmosets exhibiting germline transmission have been generated using lentiviral vectors^15–17^. Furthermore, generation of Interleukin-2 receptor subunit common gamma KO marmosets exhibiting immunodeficiency, created via introducing ZFN and TALENs into marmoset embryos has also been reported^14^. However, the efficacy of CRISPR/Cas9 in marmoset embryos has not been investigated extensively.

Host gene modification via CRISPR/Cas9 is limited by a conserved dinucleotide-containing protospacer adjacent motif (PAM) sequence, which leads to Cas9 activation^18^. Currently various Cas9 endonucleases have been developed which recognize different PAM sequences derived from several microbacteria such as *Streptococcus pyogenes* (SpCas9)^11,19^, *Staphylococcus aureus* (SaCas9)^20^ and *Francisella novicida*^21–23^. A recent study has reported that PAM recognition sequence may change via Cas9 modification^24,25^. Furthermore, a new CRISPR/Cas9 system that substitutes a single nucleotide of a specific part of a targeted gene, without involving a DSB of the host genome, has been reported^26,27^. Although its target sequence is limited by PAM, the CRISPR/Cas9 system is widely used due to ease of introduction, reasonable cost, and high efficacy for gene modification. Therefore, the applicability of this system is rapidly gaining acceptance.

In this study, the activities of CRISPR/Cas9 against two target genes, c-*kit* and *Shank3*, in marmoset embryos were investigated with particular reference to its potential for creating the marmoset target gene KO/KI models. More specifically, gene modification activity and mosaicism frequency of CRISPR/Cas9 related to c-*kit* and *Shank3* and their mosaic mutation rates in marmoset embryos were investigated.

The proto-oncogene, *c-kit*, encodes the receptor tyrosine kinase (KIT) of stem cell factor. The KIT receptor consists of an extracellular domain, containing 5 immunoglobulin-like domains, transmembrane domains, and an intracellular membrane domain carrying two kinase domains I/II^28^. In humans and mice, KIT plays important roles in the proliferation and differentiation of hematopoietic stem cells, germ stem cells, and melanocytes^28–30^. Based on this knowledge, marmoset c-*kit* gene mutants would be expected to exhibit phenotype(s) such as anaemia, infertility, and white or white spot coat colour. Therefore, the c-*kit* mutant marmosets are ideal models for studying the development of hematopoietic cells, germ cells, and melanocytes. For this reason, c-*kit* was selected as a target for CRISPR/Cas9 mediated gene modification in this study. In order to assess CRISPR/Cas9-mediated target gene KI modification, ssODNs that carry the *W*^37^ mutation, which results in an amino acid substitution on exon 11 (*W*^37^) of marmoset c-*kit*, was introduced to initiate mutation.

*Shank3*, a member of the SHANK family, encodes postsynaptic, multidomain scaffold proteins, and connects neurotransmitter receptors, ion channels and certain membrane proteins to G-protein-coupled signaling pathways and the actin cytoskeleton^31,32^. The *Shank3* mutation is strongly associated with autism and schizophrenia in humans^33–37^ and a guanine insertion at position 1227 in *Shank3* caused a frameshift mutation, yielding truncated SHANK3^33^. This mutation has been reported in patients exhibiting characteristics of autism such as severe speech impairment and mental retardation^33^. Further, *Shank3* mutations have been identified among patients, such as Q312R^36^ and R656H^37^, resulting from point mutations, and deletion of G440-P446^38^. *Shank3* mutant mice displayed differences in comparison with wild type mice in a behavioral study, such as impairment of juvenile social interactions during growth^39^, however, the mice harboring heterozygous mutations in *Shank3* exhibited by autistic patients did not readily show a phenotype associated with autism^39^. This finding suggests that it is difficult to completely replicate psychiatric disorders including autism in mice because of difference in species. NHP models are suitable to understand the pathomechanisms of autism because the NHP model displays complex cognitive functions and social behavior. In particular, social behavior within marmoset family is similar to that of humans^40^; therefore, marmosets are expected to be a suitable model for psychiatric disorders. In this study, *Shank3* was selected as a target gene for gene modification, and the efficacy of CRISPR/Cas9 against *Shank3* was examined towards the creation of marmoset autism models via *Shank3* KO.

To our knowledge, this is the first study to investigate the efficacy of target gene KI/KO and the rate of mosaicism associated with CRISPR/Cas9 in marmoset embryos.

## Results

### Validation of sgRNAs for target gene alteration rates in marmoset fibroblast cells

**Figure 1:**
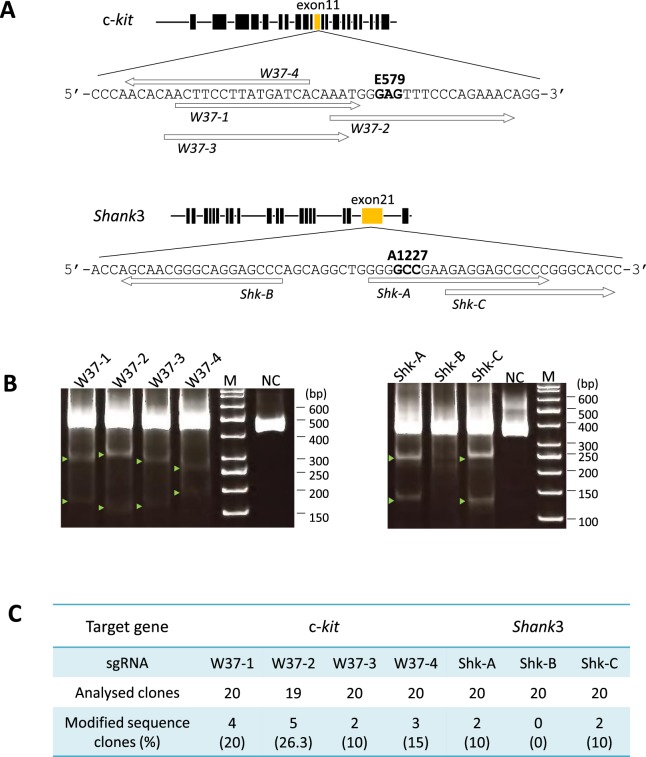
Validation of each sgRNA sequence using marmoset fibroblast cells. (**A**) Schema of linearized target genes including exons (black boxes) and single-guide RNA (sgRNA) positions (white arrows). The marmoset c-*kit* gene (top) is located on chromosome 3 and contains 21 exons. 579 glutamic acid (E579, GAG) is located on exon 11 (yellow box). Marmoset *Shank3* is located on chromosome 1 and contains 22 exons and 1227 alanine (A1227, GCC) is located on exon 21 (yellow box). (**B**) Cleavage activities of each sgRNA in marmoset fibroblast cells. The target gene modification by each sgRNA with hCas9 was examined using marmoset fibroblast cells and CEL-1 assay to confirm cleavage activity of designed sgRNAs. Green arrowheads indicate shifted bands compared to negative control, indicating target gene modification by CEL-1 assay. M; size marker, NC; negative control – PCR product obtained using wild-type marmoset tissue as a PCR template. (**C**) Rate of modified sequence clones in each target gene by subcloning and sequence analysis using PCR products of marmoset fibroblast cells.

In order to evaluate the efficacy of single guide RNA (sgRNA) in modifying the target c-*kit*, each of 4 sgRNAs, W37-1, W37-2, W37-3 and W37-4, were introduced into marmoset fibroblast cells and PCRs were performed (Fig. 1A and Supplementary Table 1). PCR primers and conditions used are indicated (Supplementary Tables 2 and 3). Target gene modification activities of the sgRNAs were validated via PCR product band densities of TBE gel electrophoresis in the CEL-1 assay. All sgRNAs against c-*kit* modified the target gene, and the rates of gene modification were 13.7%, 14.9%, 12.2%, and 9.5%, respectively (Fig. 1B). The efficacy of target gene modification was analyzed in detail using sub-cloned and sequenced of PCR products. Results of the sequence analysis indicated insertion and deletion (indel) at c-*kit* into the genome of marmoset fibroblast cells. The target gene modification rates of a total of 79 sub-clones from W37-1, -2, -3, and -4 sgRNAs were 20% (4/20 clones), 26% (5/19 clones), 10% (2/20 clones) and 15% (3/20 clones), respectively (Fig. 1C, Supplementary Table 4). There was no significant difference between these rates by Fisher’s exact test. Among these 79 clones, 11.4% showed deletion and 6.3% indicated insertion of c-*kit* (Supplementary Table 4).

The 3 sgRNAs against *Shank3*, Shk-A, Shk-B and Shk-C, were also evaluated for gene modification efficiency (Fig. 1A). Band density results of CEL-1 assay indicated that 29.3%, 0.9%, and 27.3% of cells underwent gene modification, respectively (Fig. 1B). The sequence analysis of sub-clones indicated that *Shank3* modification rates of Shk-A, -B and -C were 10% (2/20 clones), 0% (0/20 clones) and 10% (2/20 clones), respectively (Fig. 1C, Supplementary Table 5). Results of both sequence as well as target gene modification rate analyses did not indicate significant differences among the sgRNAs introduced into marmoset fibroblasts. Due to the absence of a significant difference between the target gene modification rates of the sgRNAs, the sgRNAs, W37-1 or Shk-A, were used for subsequent experiments as their sequences were the closest to the target amino acid site in each target gene.

### Target gene modification by CRISPR/Cas9 in marmoset embryos

**Figure 2:**
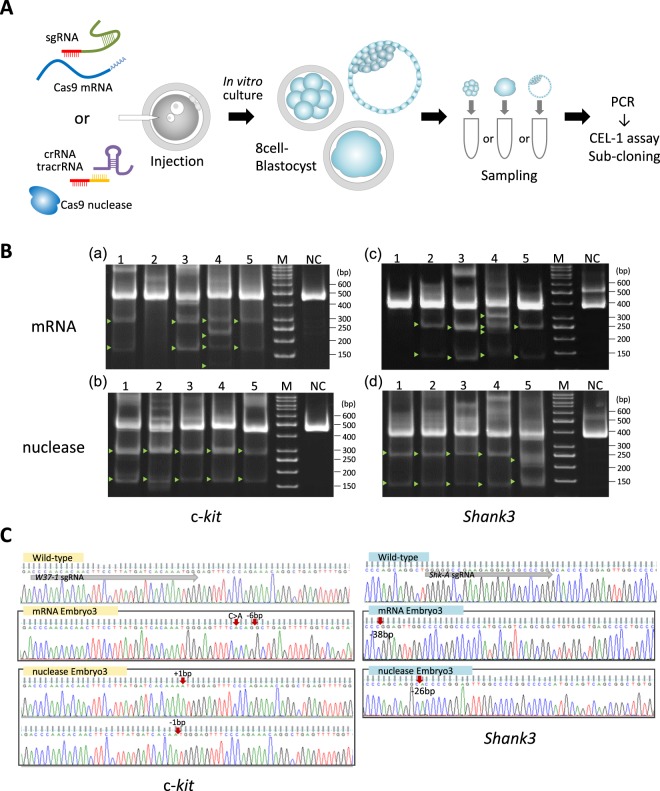
Validation of CRISPR/Cas9 cleavage activity in marmoset embryo. (**A**) Flowchart of target gene modification validation using CRISPR/Cas9 injected whole embryos. The embryos were cultured *in vitro*, and collected into PCR tubes directly after zona pellucida removal. Subsequently, PCR was performed on the collected embryos. (**B**) A portion of the CEL-1 assay result using PCR product obtained from CRISPR/Cas9 injected embryos. Upper panels show the results of c-*kit* targeted CRISPR/mRNA (mRNA) injected embryos (a), and CRISPR/nuclease (nuclease) injected embryos (b). Lower panels show the results of *Shank3* targeted CRISPR/Cas9 injected embryos (c,d). Lanes 1–5 in all panels contain Cel-1 nuclease digested DNA of PCR products obtained from each CRISPR/Cas9 injected embryo. M; size marker, NC; negative control – PCR product obtained using wild-type marmoset tissue as a PCR template. (**C**) Representative Sanger sequencing chromatograms of sub-clones derived from CRISPR/Cas9-injected embryos. Left panels show the results of sub-clones obtained from c-*kit*-targeted CRISPR/mRNA (mRNA Embryo3) or CRISPR/nuclease (nuclease Embryo3)-injected embryos. Right panels show the results of sub-clones obtained from *Shank3*-targeted CRISPR/Cas9-injected embryos (mRNA Embryo3 and nuclease Embryo3). This figure correlates with embryo samples listed in Supplemental Table 7 (c-*kit*) and 8 (*Shank3*). The top of each panel exhibits wild-type sequences of each target gene. Gray arrow; sgRNA sequence, red arrow; the position of indel or substitution mutations in each target gene.

Next, target gene modification rates of the selected sgRNAs and hCas9 mRNA (CRISPR/mRNA), as well as a combination of crRNA, tracrRNA and Cas9 nuclease (CRISPR/nuclease) were investigated in marmoset embryos (Fig. 2A). Nine out of 20 embryos injected with W37-1 CRISPR/mRNA, which developed into the 8 cell-stage or later, were analyzed (Supplementary Table 6). Seven out of 9 embryos (78%) were found to exhibit c-*kit* gene modification via CEL-1 assays (Table 1). A portion of the CEL-1 assay results, and representative Sanger sequencing chromatograms obtained from the analysis of sub-cloned sequences are shown (Fig. 2B,C). To investigate c-*kit* modification rates of CRISPR/nuclease, 25 embryos were microinjected with CRISPR/nuclease, and 15 that developed beyond the 8 cell-stage were analyzed. Among these, 9 embryos, which were successfully analyzed via CEL-1 assays, showed 100% target gene modification rate (Table 1).

**Table 1:** Rate of target gene modification by CRISPR/Cas9 in marmoset embryos.

Injected materials	Target gene	No. of embryos (%)		
		Injected	Analysed	Modified
mRNA	c-*kit*	20	9	7 (77.8)
	*Shank3*	22	5	4 (80)
nuclease	c-*kit*	25	9	9 (100)
	*Shank3*	26	12	12 (100)

The mixture of Shk-A and CRISPR/mRNA mixture was injected into 22 marmoset embryos, and 6 survived long enough to reach late embryo stages (Supplementary Table 6). Of these, 5 were successfully analyzed and 4 (80%) embryos exhibited indel modified sequences in *Shank3* (Table 1 and Fig. 2B). Of 26 embryos injected with CRISPR/nuclease mixture against *Shank3*, 14 developed beyond the 8-cell stage and 12 of them were analyzed. All 12 (100%) embryos showed modification of *Shank3* (Table 1). Part of the results from the CEL-1 assay of embryos injected with CRISPR/nuclease against *Shank3* and representative Sanger sequencing chromatograms of sub-clones are shown (Fig. 2B,C). No significant difference between the target gene modification rates of CRISPR/mRNA injection and CRISPR/nuclease was evident by Fisher’s exact test.

In order to confirm the sequence of target gene modifications such as indels, 3 randomly selected whole embryo (8 cell – Blastocyst stage) PCR samples were sub-cloned and sequence analyzed. When c-*kit* was targeted, 66.7% (insertion 20% and deletion 46.7%) and 93.9% (insertion 49.0% and deletion 44.9%) of the clones obtained from CRISPR/mRNA injected embryos and CRISPR/nuclease injected embryos, respectively, showed indel modification of the target gene (Table 2, Supplementary Table 7). When *Shank3* was targeted, 79.1%, and 96% of CRISPR/mRNA and CRISPR/nuclease injected embryos, respectively, showed deletion mutation clones (Table 2, Supplementary Table 8). The results of these sequence analyses indicated that a significantly higher number of CRISPR/nuclease injected embryos exhibited target gene modification compared to those of CRISPR/mRNA (Fisher’s exact test, *p* < 0.01).

**Table 2:** Sub-clone sequence analysis of split blastomeres.

Injected materials	Target gene	No. of clones (%)		
		Intact sequence	Indel sequence	Total
mRNA	c-*kit*	15 (33.3)	30 (66.7)	45
	*Shank3*	9 (20.1)	34 (79.1)	43
nuclease	c-*kit*	3 (6.1)	46 (93.9)	49
	*Shank3*	2 (4.0)	48 (96.0)	50

### Mosaic rate estimation by single blastomere analysis

**Figure 3:**
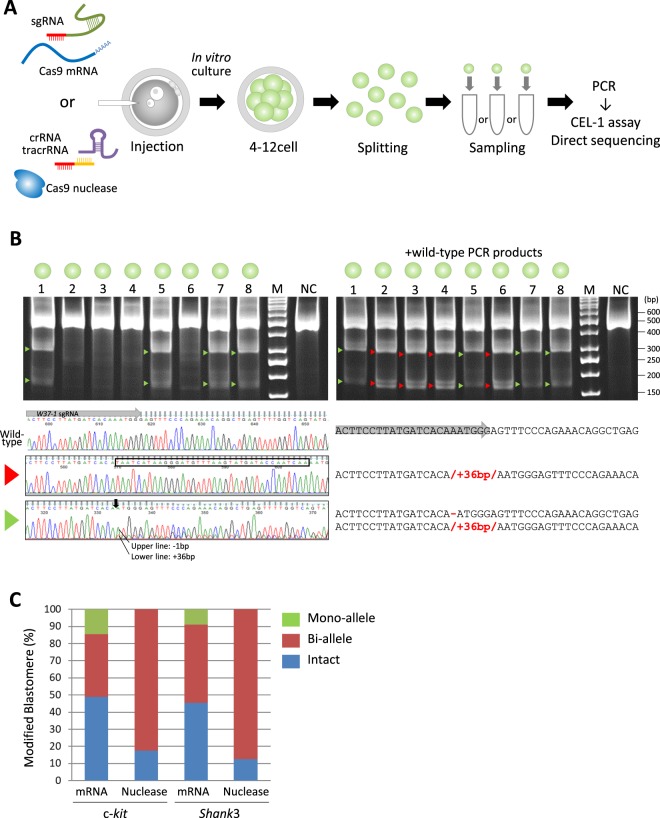
Blastomere analysis. (**A**) Flowchart used for each blastomere obtained from CRISPR/Cas9 injected embryos. The embryos were cultured, the zona pellucida was removed, and blastomeres were split. Each blastomere was collected into a PCR tube, and used for PCR analysis. (**B**) The CEL-1 assay of blastomeres from the embryo containing the c-*kit* gene modified by CRISPR/nuclease injection (Embryo2, Supplementary Table 11) in upper panels. Left panel shows the results of the CEL-1 assay of each blastomere, and right panel shows the result using a mixture of PCR products obtained from blastomere and wild-type tissues to detect homozygotic modification. Arrowheads indicate heterozygotic modification (green) and homozygotic modification (red) in the target gene. Lane 1–8 in both panels contain Cel-1 nuclease digested DNA obtained from PCR products of each blastomere. M; size marker, NC; negative control – PCR product obtained using wild-type marmoset tissue as a PCR template. Lower panels show the Sanger sequencing chromatograms (left of lower panels) and the sequences (right of lower panels) of these blastomeres. Both top lines indicate wild-type sequences of target genes. Red or green arrowheads of the left end of the lower panel are correlated with the results of the CEL-1 assay in the upper panels. Black box; inserted sequence into c-*kit*, black arrow; position of the 1-bp deletion modification, gray arrow; sgRNA sequence. (**C**) Quantitative results of target gene modification obtained from blastomere analyses. The graph shows the percentage of blastomeres containing intact (blue), bi-allelic modifications (red) and mono-allelic modifications (green) for each injection condition and target gene.

To estimate the incidence of incomplete modification such as mosaic mutation, target gene modifications in each blastomere of CRISPR/mRNA and CRISPR/nuclease injected embryos were investigated (Fig. 3A). Five days following CRISPR/mRNAs or CRISPR/nuclease injection, embryos that reached the 5- to 16-cell stage were divided into single blastomeres and each blastomere was subjected to CEL-1 assay and analyzed for gene modification via sequence analysis (Fig. 3B). Sequence analyses revealed that among the CRISPR/mRNA injected embryos, the target gene in 51.2% (c-*kit*) and 54.5% (*Shank3*) of blastomeres was modified (Table 3 and Supplementary Table 9). Of these modified blastomeres, bi-allelic modifications were found in 36.6% and 45.5% for c-*kit* and *Shank*3, respectively, and mono-allelic modifications were 14.6% and 9.1% for c-*kit* and *Shank*3, respectively (Fig. 3C, Table 3). Furthermore, all embryos injected with CRISPR/mRNA against c-*kit* or *Shank3* carried more than 2 patterns of different sequence blastomeres, except for a wild type gene embryo injected with CRISPR/mRNA against *Shank3* (Supplementary Tables 10 and 11). These sequence analyses indicated that all embryos injected with CRISPR/mRNA against c-*kit* exhibited mosaicism. on the other hand, 4 out of 5 (80%) embryos injected with CRISPR/mRNA against *Shank3* showed mosaicism, and one displayed no modification (Table 3, Supplementary Table 9).

**Table 3:** Sequence analysis of blastomeres after CRISPR/Cas injection into marmoset embryos.

Injected materials	Target gene	No. of analysed		No. of modified blastomeres (%)			No. of intact blastomeres (%)
		embryos	blastomeres	Bi-allele	Mono-allele	Total	
mRNA	c-*kit*	5	41	15 (36.6)	6 (14.6)	21 (51.2)	20 (48.8)
	*Shank3*	5	33	15 (45.5)	3 (9.1)	18 (54.5)	15 (45.5)
nuclease	c-*kit*	5	40	33 (82.5)	0 (0)	33 (82.5)	7 (17.5)
	*Shank3*	5	32	28 (87.5)	0 (0)	28 (87.5)	4 (12.5)

Of the CRISPR/nuclease injections, 82.5% of blastomeres from embryos targeted to c-*kit* and 87.5% of blastomeres from embryos targeted to *Shank3* exhibited target gene modification and all modified blastomeres carried bi-allelic mutations (Fig. 3C, Table 3). Bi-allelic mutations rates were significantly higher in CRISPR/nuclease injected embryos compared to those that received CRISPR/mRNAs injections (Fisher’s exact test, *p* < 0.01). Among these bi-allelic mutated genes, all (100%) embryos targeted to c-*kit* and 40% of embryos targeted to *Shank3* showed more than 2 patterns of mutated sequences, indicating that these were mosaic embryos. Although comparison of the numbers of modified gene patterns (Supplementary Table 12) suggest that the mosaic mutation rates in the CRISPR/mRNA and the CRISPR/nuclease injected embryos did not show significant differences, CRISPR/nuclease showed a tendency to reduce the mosaic rate.

### Efficiency of c-*kit* Knock-in

**Figure 4:**
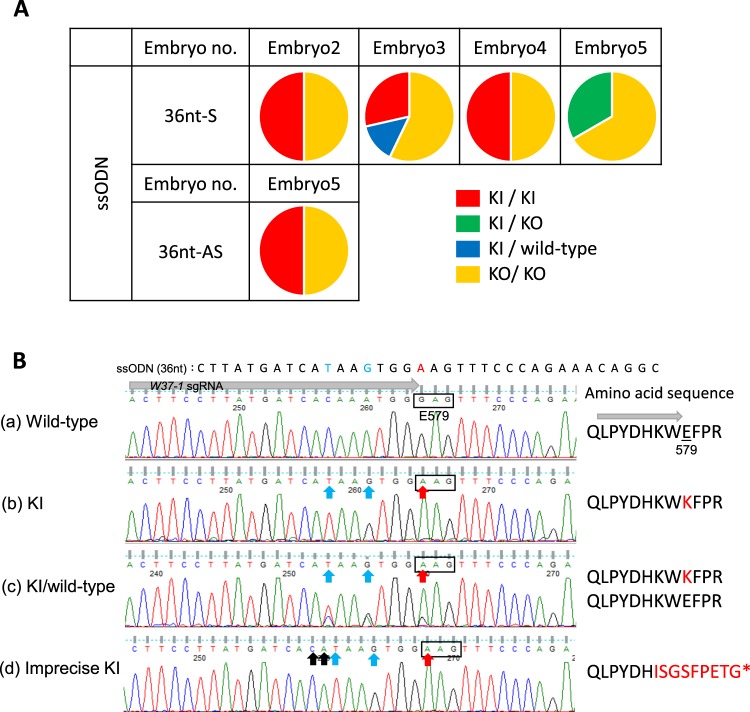
c-*kit* gene targeted Knock-in analysis by single blastomeres. (**A**) The ratio of each modified blastomere of 36 nt ssODN and CRISPR/nuclease injection KI embryos is shown. Circles represent an injected embryo in each condition, and each color indicates the blastomere sequence. Red indicates a precise KI homozygous blastomere, blue indicates heterozygous blastomere of precise KI and wild type, green represents a heterozygous precise KI and KO (indel) mutation blastomere, and yellow indicates homozygous or heterozygous KO blastomeres. Embryo number (no) s in this illustration are correlated to the Embryo no. in Supplementary Tables 14 and 15. KI: knock-in, KO: knock-out. (**B**) Representative Sanger sequencing chromatograms of modified c-*kit* and putative amino acid sequences derived from blastomere sequence analyses of c-*kit* in the KI experiment correlate with the blastomere samples listed in Supplemental Table 15. (a) Wild-type blastomere (Embryo 1, 36nt-AS and CRISPR/nuclease injection). (b) KI blastomere (Embryo 2, 36nt-S and CRISPR/nuclease injection). Blue and red arrows indicate knock-in of donor ssODN, red arrows and letters indicate mutations contributing to E579K mutant (*W*^37^). (c) KI/wild-type heterozygous blastomere (Embryo 3, 36nt-S and CRISPR/nuclease injection). (d) Imprecise KI blastomere (Embryo 1, 36nt-S and CRISPR/nuclease injection). The top sequences indicate the KI donor ssODN. The insertion (black arrows) caused a frameshift mutation and introduced a stop codon (*).

To implement target gene KI at exon 11 in marmoset c-*kit* corresponding to the *W*^37^ mutation, efficacy of target gene KI was investigated. Four single-stranded oligodeoxynucleotides (ssODN) of different lengths (36 and 100 nucleotides (nt)) and strand types (sense and anti-sense), were used for this study (Supplementary Table 13). Mixtures of CRISPR/mRNA or CRISPR/nuclease and ssODN were injected into the pronucleus and cytoplasm of marmoset embryos. Following injection, the embryos were cultured until they reached the 12 cell-stage. Subsequently, direct sequencing analyses were performed using single blastomeres to identify the most efficient ssODN knocked into the target sequence. Precise target gene KI was observed when the 36 nt length ssODN (as donor DNA) and CRISPR/nuclease were injected into embryos (Supplementary Tables 14 and 15). When 36 nt sense ssODN and CRISPR/nuclease mixture was injected into 32 embryos, 4 out of 13 analyzed embryos (30.8%) were found to contain precise KI blastomeres (Supplementary Table 16). By contrast, when 36 nt antisense ssODN was used as KI donor DNA, 12 out of 29 injected embryos were analyzed and only 1 out of 12 analyzed embryos (8.3%) carried precise KI blastomeres (Supplementary Table 16). All injected embryos including KI embryos carried the mosaic mutations (Fig. 4A, Table 4). Some imprecise KI blastomeres were also observed among these embryos (Fig. 4B(d)), Table 4 and Supplementary Table 15). Following injection of sense 36 nt ssODN, precise KI blastomere rates in each embryo were 50%, 42.9%, 50%, and 33.3% (Supplementary Table 14), corresponding to 31.6% of analyzed blastomeres (Table 4). Precise KI efficiencies in the blastomeres were significantly higher for the sense 36 nt ssODN (*p* < 0.01, Fisher’s exact test, Ryan’s multiple comparison).

**Table 4:** Efficiencies of the c-*kit* gene KI by CRISPR/Cas9.

Injected materials	ssODN		No. of analysed		No. of intact blastomeres(%)	No. of modified blastomeres (%)		
						Indel	KI	
	length	strand	embryos	blastomeres			Imprecise	precise
mRNA	36 nt	sense	5	49	17 (34.7)	32 (65.3)	0 (0)	0 (0)
		anti-sense	5	37	11 (29.7)	25 (67.6)	1 (2.7)	0 (0)
	100 nt	sense	5	36	15 (41.7)	21 (58.3)	0 (0)	0 (0)
		anti-sense	5	39	19 (48.7)	20 (51.3)	0 (0)	0 (0)
nuclease	36 nt	sense	5	38	0 (0)	24 (63.2)	2 (5.3)	12 (31.6)
		anti-sense	5	42	5 (11.9)	17 (40.5)	17 (40.5)	3 (7.1)
	100 nt	sense	5	39	37 (94.9)	2 (5.1)	0 (0)	0 (0)
		anti-sense	5	34	29 (85.3)	5 (14.7)	0 (0)	0 (0)

In these analyses, 20 imprecise KI blastomeres were also detected (Table 4, Supplementary Table 15) via sequence analysis (Supplementary Table 16) by 36 nt sense and anti-sense ssODN. Interestingly, precise KI and imprecise KI were not observed in the same embryo. Imprecise KI creates a stop codon on the sixth amino acid downstream of c-*kit*, E579, subsequently causing a c-*kit* KO mutation (Fig. 4B).

#### Prediction of potential off-target sites

Potential off-target sites of sgRNA were predicted using an online tool CRISPOR (http://crispor.org). Off-target sites including 3 or less mismatches in comparison with each sgRNA sequence were analyzed. The results are shown in Supplementary Table 17. Off-target sites of 0, 1, and 2 mismatches with NGG recognition were not detected in all sgRNAs; however, 3 mismatched off-target candidates were detected for each sgRNA (Supplementary Table 17). We evaluated the off-target score, which is probability of presenting as unexpected target sites, were determined on the basis of mismatch positions in sgRNA sequences. In this score, a value “1” indicates a perfect match with sgRNA, indicating high potential as an off-target site, and a value of “0” indicates no potential as an off-target site^41^. A score greater than 0.2 are thought to be an off-target site^41^. The number of off-target sites with scores greater than 0.2 were 4, 6, 3, and 4 for W37-1, -2, -3, and -4 sgRNAs, respectively. For *Shank3* sgRNAs, 6, 8, and 4 off-target candidates exhibited an off-target score greater than 0.2 for Shk-A, -B and -C sgRNAs, respectively.

## Discussion

In this study, the modification efficiency of CRISPR/Cas9 against two target genes, c-*kit* and *Shank3*, were investigated in marmoset embryos. Furthermore, target gene modifications in blastomeres were analyzed to verify mosaicism in embryos following CRISPR/mRNA or CRISPR/nuclease injection in order to predict whether first generation target gene KO/KI animals may display objective phenotypes. Although CRISPR/nuclease showed 100% modification in marmoset whole embryos for both target genes, modification rates determined via CEL-1 assay were not significantly different compared to those for CRISPR/mRNA due to both artificial nuclease injections exhibiting a high degree of efficiency in target gene modification (Table 1). Sequence analysis of subcloned PCR products from whole embryos indicated that mosaic modifications of target genes were observed in both CRISPR/mRNA and CRISPR/nuclease injected embryos. Although CRISPR/nuclease displayed a tendency to reduced mosaic modification, the mosaic modification rates between these two conditions were not significantly different (Fig. 3C, Table 3 and Supplementary Table 12) and there was no significant difference between the target genes or sgRNA.

CRISPR/Cas9-induced mosaic mutations have been reported in several species^13,42,43^. In mosaic animals with intact genes as well as target gene KO/KI mutations, target gene KO/KI mutated gene phenotype(s) may be rescued by intact genes, causing them to revert to wild type phenotypes. In mice, such displays are not significant because a second generation is produced within 2–3 months, as well, second-generation animals will be non-mosaic and show objective phenotypes. However, this is not applicable for NHPs, which require more than 2 to 5 years to commence reproduction; even marmosets require 2 to 3 years to produce a second generation. Therefore, it may be preferable for target gene KO/KI NHP models to not carry the intact target gene. Mosaic alteration was not observed when platinum TALEN was used to KO the interleukin receptor common gamma gene (*il2rg*), and all male animals exhibited immune deficient phenotypes^14^. In contrast, *il2rg* heterozygous (WT/indel) modified female marmoset exhibited the wild type phenotype because the intact target gene rescued the mutant phenotype^14^. Recent reports indicated that Cas9 nuclease together with crRNA and tracrRNA showed a high rate of KI (46%) compared with Cas9 mRNA (13%) in mice, where 51.4% of F1 progeny showed KI genotype, suggesting a low level of mosaic mutation in founder generations^44^. Since CRISPR/nuclease does not require translation from mRNA, it causes rapid DSB and reduces mosaicism in embryos. Because of these reasons, and due to its tendency for reducing mosaic alterations as well as retaining intact gene, CRISPR/nuclease appeared to be more suitable for producing target gene KO marmosets. On the other hand, Cas9 nuclease injected marmoset embryos also showed several patterns of target gene modification among in the blastomeres. For example, there were several blastomeres that carrying an identical indel sequence in one allele but a different indel sequence in another allele. This may be explained via CRISPR/Cas9-mediated DSB persisting from one-cell stage until cleavage stage in embryos. As a result, Cas9 nuclease generated high rates of bi-allelic target gene KO when injected into embryos, which may be lethal to embryos due to loss of gene function. Such a high rate of bi-allelic target gene KO in the embryos requires development of new methodology to alter a target gene. Tu *et al*. reported that *Cas9*, when combined with ubiquitin-proteasome signal, reduced mosaicism of target gene modification in cynomolgus monkeys^45^. This newly devised CRISPR/Cas9 led to early degradation of Cas9, and therefore CRISPR/Cas9 was unable to function in developed monkey embryos following injection. Thus, it is possible that Cas9 incorporating a ubiquitin-proteasome signal may reduce mosaic mutation in the marmoset embryo and generate homogeneous target gene modified animals. Furthermore, in order to reduce mosaic mutations in human embryos, Ma *et al*. reported that following CRISPR/Cas9 co-injection with sperm into M-phase oocytes, 98.2% of embryos showed non-mosaic alterations upon correction of *MYBPC3* mutations in human embryos^46^. Reasons of this high rate of uniform embryos seems to be acquired not only during M-phase injection, but was also because sgRNA was designed to specifically target mutant *MYBPC3*, but not the wild type target gene. Furthermore, the *MYBPC3* mutant specific sgRNA appeared to repair the *MYBPC3* deletion mutation via homology-directed repair using the maternal wild-type allele as a template instead of the injected ssODNs^46^. Although using mutant specific sgRNA is difficult to induce mutant gene into wild type target gene unless if single nucleotide polymorphism exists in target gene, Cas9 combined with a ubiquitin-proteasome signal and CRISPR/Cas9 injection into M-phase oocytes approaches may useful for target gene modification and mosaic mutation reduction in marmoset embryos, and may result in the efficient generation of genetically modified marmosets exhibiting phenotypes.

The current study also attempted to substitute 579 glutamic acid (GAG) of marmoset c-KIT to lysine (AAG), which is known to produce *W*^37^ mutant mice^47,48^, by target gene KI using CRISPR/Cas9 and donor ssODN. Single blastomere analyses of injected embryos revealed that precise KI mutations were effectively modified when the 36 nt sense ssODN was used as a donor and precise KI mutation was not observed when CRISPR/mRNA was used (Supplementary Table 14). Furthermore, although CRISPR/nuclease showed high efficiency of target gene KI under specific conditions, all these embryos also contained c-*kit* gene KO blastomeres. From the sequence, these embryos would display *W* phenotypes but not phenotypes identical to *W*^37^. Several blastomeres exhibited imprecise KI mutations in the form of cytosine and adenine insertions on the 5′ end of the ssODN sequence (Fig. 4B, Supplementary Table 15). KI strategy using CRISPR/Cas9 with ssODN has been reported in rat models^49^. Yoshimi *et al*. presumed that a DSB in the host genome may cause a 5′ to 3′ end resection due to 5′ exonuclease activity, and that donor ssODN was also similarly resected, consequently resulting in imprecise KI^49^. They further speculated the possibility of triggering unexpected genetic modifications such as indel when ssODN is incorporated via microhomology-mediated end joining (MMEJ). Based on the findings of the above study, it was speculated that the putative cause of our erroneous mutation may be an inappropriate MMEJ event. An accurate reason for the generation of efficient precise KI with sense strand-ssODN is unclear, but our data suggested that a MMEJ error is unlikely to occur when CRISPR/nuclease and sense-strand ssODN are used in c-*kit* exon 11 KI modification. When long-length-ssODN (100 nt) and CRISPR/nuclease were used, neither KI of c-*kit* at exon 11, nor KO of c-*kit* were frequently observed (Fig. 4 and Supplementary Table 14). In this system, inefficient target gene cleavage may result in a reduced target gene KI event. Therefore, we speculated that the co-injected donor ssOND length may affect cleavage activity of CRISPR/Cas9.

The potential off-target sites were predicted using a web tool and 50 off-target sites for c-*kit* and 46 off-target sites of *Shank3* were observed. These potential off-target sites presented in introns or intergenic regions of marmoset chromosomes, except for several off-target sites of W37-2, W37-4, and Shk-C sgRNA. In this study, W37-1 and Shk-A sgRNA were used to modify embryonic genes in order to establish target gene KI/KO model marmosets. Therefore, the selected sgRNA seemed suitable for establishing KI/KO marmoset models for these target genes.

This study showed that CRISPR/Cas9 efficiently modified the target genes in marmoset embryos. However, the resulting gene alteration would be a mosaic modification. Furthermore, this mosaic modification seemed to be caused by long term activity of Cas9 nuclease in the marmoset embryos, which led to bi-allelic modification of target gene KO, resulting in complete loss of gene function. This loss of gene function may be lethal to the embryo that is developing into the model marmosets. Therefore, selecting CRISPR/mRNA or CRISPR/nuclease is important for creating marmoset target gene KO models. Furthermore, although CRISPR/nuclease showed a high degree of efficiency in target gene KI under specific conditions, the embryos also contained target gene KO blastomeres. This indicates that in order to obtain precise KI animals without inaccurately modified blastomeres, extensive examination of the target gene KI conditions would be required. Well-defined preliminary studies will pave the way to obtain objective NHP models and reduce euthanasia of failed gene editing animals via CRISPR/Cas9.

## Methods

### Construction of guideRNA and Cas9 expression vectors

To construct the sgRNA transcription vectors, sense and antisense strand oligonucleotides of target sequences (synthesized by FASMAC Co., Ltd (Kanagawa, Japan)) were phosphorylated using T4 Polynucleotide Kinase (New England Biolabs, M0201) according to manufacturer’s recommendations. Complementary pairs of phosphorylated oligonucleotides were incubated at 95 °C for 2 min, cooled down to 25 °C to anneal at −0.1 °C per second. Annealed DNA was inserted into *Bsa*I site of U6-sgRNA expression vector, which was previously described^50^ and used for validation of sgRNAs in marmoset fibroblast cells. To synthesize sgRNA mRNA for microinjection into marmoset embryos, annealed DNA was inserted into the *Bsa*I site of the DR274 vector (Addgene #41815) carrying T7 promoter for *in vitro* transcription. The oligonucleotide sequences of the sgRNAs are listed (Supplementary Table 1). Humanized Cas9 (hCas9) expression vector (pST1374-NLS-flag-linker-Cas9, Addgene #44758)^51^ containing the CMV promoter and T7 promoter was used for validation in marmoset fibroblasts cells and mRNA synthesis.

### Donor DNA

Single-stranded-oligonucleotides (ssODNs) used as donor DNA for KI experiments were designed to introduce point mutations in the marmoset c-*kit*, corresponding to *W*^37^ mutant mice. The marmoset c-*kit* sequence was obtained from the previously reported marmoset genome database^52^. In marmoset c-*kit* gene, *W*^37^ mutant is involved in exon 11 mutation. The 36 nt and 100 nt long sense strand ssODN (36 nt-S, 100 nt-S) and antisense strand ssODN (36 nt-AS, 100 nt-AS) required to introduce the mutation into the c-*kit* gene exon 11 were designed so that amino acid residue 579 was replaced with lysine (AAG) from glutamic acid (GAG) (Supplementary Table 13). All ssODNs included silent mutations to avoid recognition by CRISPR/Cas9 following KI. These ssODNs were synthesized and purified by HPLC grade (FASMAC Co., Ltd.).

### Validation of CRISPR/Cas9 activity in marmoset fibroblast cell

Marmoset fibroblast cells were seeded at 5 × 10^4^ cells per well of a 12-well culture plate with Dulbecco’s modified Eagle’s medium + GlutaMAX (Thermo Fisher Scientific, 10566-024) containing 10% FBS (Biowest), penicillin-streptomycin cocktail (Thermo Fisher Scientific, 15240-062), and cultured for 16–18 hr at 37 °C, 5% CO_2_. U6-sgRNA expression vector including the target sequence and hCas9 expression vector were transfected into the cultured cells using Lipofectamine LTX Reagent with PLUS Reagent (Thermo Fisher Scientific, 15338-100) and Opti-MEM (GIBCO, 31985-062), according to the manufacturer’s protocol. Plasmid DNA concentrations were as follows; 0.5 µg of sgRNA expression vector and 0.5 µg of hCas9 expression vector were placed in a well. After transfection, cells were incubated at 37 °C, 5% CO_2_ for 2 days, harvested and genome DNA was extracted from harvested cells using GenElute Mammalian DNA prep Kit in accordance with the manufacturer’s instructions (Sigma, G1N70-1KT). Extracted genomic DNA was used to detect target gene modification as described below. These examinations involved 3 trials for each sgRNA, and modification efficacy was calculated using the densities of mutation-suggesting-band of CEL-1 assay using ImageJ software. The intensity of each band of the CEL-1 assay was calculated using ImageJ, and the percentage of modified cells was determined on the basis of the band intensities.

### Animals, oocyte collection and *In Vitro* fertilization

All animal experiments were approved by the Institutional Animal Care and Use Committee of the Central Institute for Experimental Animals (CIEA: 11028), and performed in accordance with CIEA standard guidelines. The CIEA standard guidelines are in accordance with the guidelines for the proper conduct of animal experiments determined by the Science Council of Japan. Adult marmosets (2–4.5 years; body weight 300–450 g) used in this study were purchased from an experimental animal breeding company (CLEA Japan, Inc., Tokyo, Japan). Oocyte collection and pronuclear stage embryo production were performed via *in vitro* fertilization (IVF) as previously described^14,53^. Briefly, marmoset germinal vesicle-stage (GV) oocytes were surgically collected from ovarian-stimulated female marmosets whose ovarian cycles were monitored based on plasma progesterone levels.

Follicular stimulation was performed as described below. Twenty-five IU of human follicle-stimulating hormone (hFSH, Fuji Pharma) was intramuscularly injected into female marmosets for 9 days every other day. Subsequently, 75 IU of human chorionic gonadotropin (hCG, ASKA Pharmaceutical) was intramuscularly injected 17–20 hr before surgery. GV oocytes were collected by aspiration of the ovum using a disposable syringe from the anesthetized female marmosets. Collected oocytes were matured in POM medium (Research Institute for the Functional Peptides, IFP1010P) in 5% FBS, 0.15 IU/mL of hFSH and 10 IU/mL of hCG for 27–29 hr at 37 °C with 5% CO_2_, 5% O_2_, and 90% N_2_. Sperm of wild-type male marmosets were introduced via IVF into matured oocytes. The sperm was collected from healthy male marmosets with physical stimulation and was diluted to 3.6 × 10^6^ sperm/mL in TYH medium (LSI Medience, DR01031). Matured oocytes and diluted sperm were co-incubated in TYH medium for 10–16 hr at 37 °C with 5% CO_2_, 5% O_2_, and 90% N_2_. After IVF, pronuclear stage embryos were obtained for experimental use.

### Validation of CRISPR/Cas9 activity in marmoset embryos

To validate the target gene modification by CRISPR/Cas9 in marmoset embryos, *in vitro* transcription of sgRNA was performed on DR274 vectors including each sgRNA target sequence. These vectors were linearized with *Hind*III (New England BioLabs, R0104), and purified with QIAquick Gel Extraction Kit (Qiagen, 28706). Linearized vector DNA was used as a template for mRNA synthesis using Mega shortscript T7 Transcription Kit (Ambion, AM1354), mMessage Machine T7 Ultra kit (Ambion, AM1345M) and MEGAclear kit (Ambion, AM1908). All procedures were performed according to the manufacturer’s instructions. The CRISPR/Cas9 mRNA mixture injected into embryos was composed of 50 ng/μL of sgRNA and 100 ng/μL of Cas9 mRNA. The CRISPR/Cas9 nuclease injection mixture, which was composed of 16.7 ng/μL of CRISPR RNA (crRNA), 33.3 ng/μL of transactivating crRNA (tracrRNA), 100 ng/μL of Cas9 nuclease (Integrated DNA Technologies, 1074181) and 0.1 × Tris-EDTA pH 8.0 buffer, was incubated at 37 °C for 30 min before injection. Sequences of crRNAs were identical to the sgRNA sequence (Supplementary Table 1); crRNA and tracrRNA were synthesized at FASMAC Co., Ltd. Approximately 4–8 pL of injection mixture was injected into the cytoplasm of marmoset pronuclear stage embryos using an air-pressure microinjector (FemtoJet, Eppendorf). In the KI experiments, 50 ng/μL ssODN was injected into marmoset embryo pronucleus at first, and subsequently CRISPR/Cas9 was injected into cytoplasm. After injection, embryos were cultured in Sequential Cleav medium (Origio, 83040010A) at 37 °C, with 5% CO_2_, 5% O_2_, and 90% N_2,_ until the 8-cell stage developmental phase. Subsequently, developed embryos were placed in Sequential Blast medium (Origio, 83060010 A) supplemented with 10% FBS, 2.18 mM L-glutamine (GIBCO, 25030-081) and were continuously cultured at 37 °C with 5% CO_2_, 5% O_2_, and 90% N_2_. To validate gene modification in embryos, cultured embryos were washed with PBS (−) and the zona pellucida was removed using acidified tyrode solution (Origio, 10605000). Each bared embryo was collected in 0.2 ml PCR tubes, and used directly as a PCR template to detect target gene modifications.

### Blastomere analysis

CRISPR/Cas9 injected embryos were cultured for approximately 5 days, until the 5–16 cell embryo phase was reached. Embryos were washed with PBS (−), the zona pellucida was removed. Bared embryos were then split into single blastomeres using glass capillaries and soaked in Embryo Biopsy Medium (Irvine Scientific, 90103) for 15 min at 37 °C. Each single blastomere was collected as a template in 0.2 ml PCR tubes and PCR without genome extraction was performed to detect target gene modifications.

### Detection of target gene modifications

To detect the target gene modification, nested-PCR and CEL-1 assay were performed using the following conditions. For PCR, a reaction mixture composed of 1X PCR buffer, 0.2 mM dNTPs, 0.5 mM of each primer, 2.5 U of KOD-Plus-Neo (TOYOBO, 401) and template DNA was prepared. Twenty nanograms of genomic DNA extracted from a marmoset fibroblast, a whole embryo and a blastomere, respectively, were used as PCR templates. The primer sets and PCR conditions used in this study are listed in Supplementary Tables 2 and 3. All amplified products were electrophoresed in a 1.5% agarose gel to determine whether the PCR was successful. CEL-1 assay, which enables detection of the target gene modification, was performed using a SURVEYOR mutation detection kit (Integrated DNA Technologies, 706020) according to the manufacture’s protocol. The mismatch reaction before Cel-1 nuclease digestion used 8 μL of PCR products. To detect homozygous mutations from CEL-1 assay using single blastomere analysis, 4 μL of PCR product derived from blastomere was mixed with 4 μL of amplified DNA of the target locus derived from the wild-type tissue genome, and a mismatch reaction and CEL-1 assay was carried out. The DNA solutions digested with Cel-1 nuclease were applied to a well of 10% TBE Gel (Invitrogen, EC62752BOX), and electrophoresis was performed at 170 V for 60 min. After electrophoresis, TBE gels were stained with intercalating nucleic acid stain solution (GelRed, Wako, 518-24031), which was diluted 1:10000 in distilled water and was visualized by UV irradiation. The sub-clones of the PCR products were obtained using Zero blunt TOPO PCR cloning kit (Invitrogen, 450245) for sequence analysis. Sequence analysis of sub-clones and PCR products was performed using the 3130 Genetic Analyzer.

### Prediction of potential off-target sites

Potential off-target sites were predicted using online tool, CRISPOR (http://crispor.org)^54^.

### Statistical analysis

The statistical analysis of gene modification efficiencies was performed by Fisher’s exact test. The KI-blastomere generation efficiency was evaluated by one-way analysis of variance followed by Ryan’s multiple comparison test. Differences with P-values < 0.05 were considered statistically significant.

## Supplementary information

Dataset 1

## Data Availability

All data generated or analyzed during this study are included in this published article (and its Supplementary Information Files).
