# Supplementary material for: Efficient generation of Knock-in/Knock-out marmoset embryo via CRISPR/Cas9 gene editing
Source: Sci Rep. 2019 Sep 3;9:12719. doi: 10.1038/s41598-019-49110-3 (PMC6722079; doi:10.1038/s41598-019-49110-3)
Supplement: Supplementary file 1 — Dataset 1 [file 41598_2019_49110_MOESM1_ESM.pdf]

Supplementary Information

Title

"Efficient generation of Knock-in/Knock-out marmoset embryo via CRISPR/Cas9 gene editing."

Authors

Wakako Kumita[1], Kenya Sato[1], Yasuhiro Suzuki[1], Yoko Kurotaki[1], Takeshi Harada[2, 3], Yang Zhou[4], Noriyuki Kishi[5], Kengo Sato[6], Atsu Aiba[3], Yasubumi Sakakibara[6],

Guoping Feng[4], Hideyuki Okano[5, 7, 8] and Erika Sasaki[1, 5, 8]*

[1]Central Institute for Experimental Animals, Kawasaki-shi, Kanagawa, 210-0821, Japan.

[2]Department of Molecular Biology and Biochemistry, Graduate School of Medicine, Osaka University, Suita-shi, Osaka, 565-0871, Japan

[3]Laboratory of Animal Resources, Center for Disease Biology and Integrative Medicine, Graduate School of Medicine, The University of Tokyo, Hongo, Bunkyo-ku, Tokyo, 113-8655, Japan

[4]McGovern Institute for Brain Research, Department of Brain and Cognitive Sciences, Massachusetts Institute of Technology, Cambridge, Massachusetts, 02139, USA

[5]Laboratory for Marmoset Neural Architecture, RIKEN Center for Brain Science, Wako-shi, Saitama, 351-0198, Japan

[6]Department of Biosciences and Informatics, Keio University, Yokohama-shi, Kanagawa, 223-8522, Japan

[7]Department of Physiology, Keio University School of Medicine, Shinjuku-ku, Tokyo, 160-8582, Japan

[8]Advanced Research Center, Keio University, Shinjuku-ku, Tokyo 160-8582, Japan

*Corresponding author

e-mail and telephone: esasaki@ciea.or.jp, +81-44-201-8545

Supplementary Table 1. Sequence of sgRNAs

Target	Location/Gene ID	sgRNA	5'-sequence-3'	PAM	strand
c-*kit*	Chr.3: 138848396-138932796	W37-1	ACTTCCTTATGATCACAAAT	GGG	+
	Gene ID: 100411615	W37-2	AATGGGAGTTTCCCAGAAAC	AGG	+
		W37-3	AACTTCCTTATGATCACAAA	TGG	+
		W37-4	GATCATAAGGAAGTTGTGTT	GGG	−
Shank3	Chr.1: 210201841-210277470	Shk-A	GGGGCCGAAGAGGAGCGCCC	GGG	+
	Gene ID: 100405926	Shk-B	GGGCTCCTGCCCGTTGCTGG	TGG	−
		Shk-C	AGAGGAGCGCCCGGGCACCC	CGG	+

Supplementary Table 2. Primers for PCR and sequence analysis

Target	Use	Primer	5'-sequence-3'	Product size (bp)
c-*kit*	1st PCR	forward	TGGAATTGCGGGGCTATGGCAG	745
		reverse	AACCTTCCCAAAAGCACCAGC	
	2nd PCR / sequence analysis	forward*	AAATCCAGCCCCACACCCTGTTCA	454
		reverse	ATCAGAGGAGGTGCAATTTCACAGA	
*Shank*3	1st PCR	forward	TCGACGAGCGCCTCTTGGGCACC	735
		reverse	CCCTGGTCTCCTCATCGCTA	
	2nd PCR / sequence analysis	forward*	TGTGGATGTGCAGGCCCGGGACCCAGAGC	392
		reverse	CTGGCTCTTCCTCTGAGCTGCCCTGACCTG	

*Forward primers of 2nd PCR were used for sequence analysis in each target gene.

Supplementary Table 3. PCR conditions

Target	Use	Reaction temperatures and cycles		
c-*kit*	1st and 2nd PCR	Pre-denaturation:	94°C, 2 min	
		Denaturation:	98°C, 10 sec	5 cycles
		Extension:	74°C, 30 sec	
		Denaturation:	98°C, 10 sec	5 cycles
		Extension:	72°C, 30 sec	
		Denaturation:	98°C, 10 sec	5 cycles
		Extension:	70°C, 30 sec	
		Denaturation:	98°C, 10 sec	28 cycles
		Extension:	68°C, 30 sec	
		Extension:	68°C, 7 min	
*Shank*3	1st PCR	Pre-denaturation:	94°C, 2 min	
		Denaturation:	98°C, 10 sec	5 cycles
		Extension:	74°C, 30 sec	
		Denaturation:	98°C, 10 sec	5 cycles
		Extension:	72°C, 30 sec	
		Denaturation:	98°C, 10 sec	5 cycles
		Extension:	70°C, 30 sec	
		Denaturation:	98°C, 10 sec	45 cycles
		Extension:	68°C, 30 sec	
		Extension:	68°C, 7 min	
	2nd PCR	Pre-denaturation:	94°C, 2 min	
		Denaturation:	98°C, 10 sec	5 cycles
		Extension:	74°C, 30 sec	
		Denaturation:	98°C, 10 sec	5 cycles
		Extension:	72°C, 30 sec	
		Denaturation:	98°C, 10 sec	5 cycles
		Extension:	70°C, 30 sec	
		Denaturation:	98°C, 10 sec	40 cycles
		Extension:	68°C, 30 sec	
		Extension:	68°C, 7 min	

Supplementary Table 4. Sub-cloning analysis of CRISPR/Cas9-mediated c-*kit* gene modification in marmoset fibroblast cells

sgRNA	Sequence	No. of sub-clones			Modified sequence
		Intact	Modified	Total	
Intact sequence	CCCAACACAACTTCCTTATGATCACAAATGG**GAG**TTTCCCAGAAACAGGCTGAGTTTTGG				
W37-1	CCCAACACAACTTCCTTATGATCACAAAATGGGAGTTTCCCAGAAACAGGCTGAGTTTTGG		2	20	+1bp
	CCCAACACAACTTCCTTATGATC-CAATGGGAGTTTCCCAGAAACAGGCTGAGTTTTGG		1		-1bp
	CCCAACACAACTTCCTT----------ATGGGAGTTTCCCAGAAACAGGCTGAGTTTTGG		1		-10bp
	CCCAACACAACTTCCTTATGATCACAAATGGGAGTTTCCCAGAAACAGGCTGAGTTTTGG	16			Intact
	Total (%)	16 (80)	4 (20)		
W37-2	CCCAACACAACTTCCTTATGATCACAAATGGGAGTTTCCC--AAACAGGCTGAGTTTTGG		1	19	-2bp
	CCCAACACAACTTCCTTATGATCACAAATGGGAGTTTCCCAGAA-CAGGCTGAGTTTTGG		2		-1bp
	CCCAACACAACTTCCTTATGATCACAAATGGGAGTTTCCC-------GGCTGAGTTTTGG		1		-7bp
	CCCAACACAACTTCCTTATGATCACAAATGGGAGTTT------AACAGGCTGAGTTTTGG		1		-6bp
	CCCAACACAACTTCCTTATGATCACAAATGGGAGTTTCCCAGAAACAGGCTGAGTTTTGG	14			Intact
	Total (%)	14 (73.7)	5 (26.3)		
W37-3	CCCAACACAACTTCCTTATGA-------------------------AGGCTGAGTTTTGG		1	20	-25bp
	CCCAACACAACTTCCTTATGATCACAAAATGGGAGTTTCCCAGAAACAGGCTGAGTTTTGG		1		+1bp
	CCCAACACAACTTCCTTATGATCACAAATGGGAGTTTCCCAGAAACAGGCTGAGTTTTGG	18			Intact
	Total (%)	18 (90)	2 (10)		
W37-4	CCCAAACACAACTTCCTTATGATCACAAATGGGAGTTTCCCAGAAACAGGCTGAGTTTTGG		1	20	+1bp
	CCCAAAACACAACTTCCTTATGATCACAAATGGGAGTTTCCCAGAAACAGGCTGAGTTTTGG		1		+2bp
	CCC----ACAACTTCCTTATGATCACAAATGGGAGTTTCCCAGAAACAGGCTGAGTTTTGG		1		-4bp
	CCCAACACAACTTCCTTATGATCACAAATGGGAGTTTCCCAGAAACAGGCTGAGTTTTGG	17			Intact
	Total (%)	17 (85)	3 (15)		

Bold letters showed E579 at c-*kit* gene exon11. Underlines indicated each sgRNA sequence, and red letters indicated insertion/deletion modification of target gene.

Supplementary Table 5. Sub-cloning analysis of CRISPR/Cas9-mediated *Shank*3 gene modification in marmoset fibroblast cells

sgRNA	Sequence	No. of sub-clones			Modified sequence
Intact sequence	GCCACCAGCAACGGGCAGGAGCCCAGCAGGCTGGGG**GCC**GAAGAGGAGCGCCCGGGCACCCCGGAGTT	Intact	Modified	Total	
Shk-A	GCCACCAGCAACGGGCAGGAGCCCAGCAGGCTGGGGGCGGAAGAGGAGCGCCCGGGCACCCCGGAGTT		1	20	C>G
	GCCACCAGCAACGGGCAGGAGCCCAGCAGGCTGGGGGCCGAAGACGAGCGCC------------TCTT		1		-14bp, +2bp
	GCCACCAGCAACGGGCAGGAGCCCAGCAGGCTGGGGGCCGAAGAGGAGCGCCCGGGCACCCCGGAGTT	18			Intact
	Total (%)	18 (90)	2 (10)		
Shk-B	GCCACCAGCAACGGGCAGGAGCCCAGCAGGCTGGGGGCCGAAGAGGAGCGCCCGGGCACCCCGGAGTT	20		20	Intact
	Total (%)	20 (100)	0 (0)		
Shk-C	GCCACCAGCAACGGGCAGGAGCCCAGCAGGCTGGGGGCCGAAGAGGAGCGCCCGGGCAAGGGGAATG		1	20	replacement
	GCCACCAGCAACGGGCAGGAGCCCAGCAGGCTGGGGGCCGAAGAGGAGCGCCCGGGCACCCCGGAGTT	19			
	Total (%)	19 (95)	1 (5)		

Bold letters showed *Shank3* gene A1227. Underlines indicated each sgRNA sequence, and red letters indicated insertion/deletion modification of target gene.

Supplementary Table 6. Efficiency of CRISPR/Cas9 activity in marmoset embryos

Target	Injected materials	Injected	Development			Analyzed	Modified (%) **
			Dead	1-7cell	8cell-Blast (%) *		
c-*kit*	mRNA	20	4	7	9 (45)	9	7 (77.8)
	nuclease	25	5	5	15 (60)	9	9 (100)
*Shank*3	mRNA	22	8	8	6 (27.3)	5	4 (80)
	nuclease	26	5	7	14 (53.8)	12	12 (100)

*Developmental stage of *in vitro* cultured embryos at Day11 after injection, and the efficiencies were calculated from the number of injected embryos.

**Calculated from analyzed embryos.

Supplementary Table 7. Sub-cloning analysis of CRISPR/Cas9-mediated c-*kit* gene modification in marmoset embryos

Injected materials	Embryo no.	Sequence	No. of sub-clones				Modified sequence
Intact sequence		CCAACACAACTTCCTTATGATCACAAATGG**GAG**TTTCCCAGAAACAGGCTGAGTTTTGG	Intact	Ins	Del	Total	
mRNA	Embryo 1	CCAACACAACTTCCTTATGATCACA--TGGGAGTTTCCCAGAAACAGGCTGAGTTTTGG			4	13	-2bp
		CCAACACAACTTCCTTATGATCACAA-TGGGAGTTTCCCAGAAACAGGCTGAGTTTTGG			3		-1bp
		CCAACACAACTTCCTTATGATCACAAATGGGAGTTTCCCAGAAACAGGCTGAGTTTTGG	6				
	Embryo 2	CCAACACAACTTCCTTATGATCACAAATGGGAGTTTCCCAAAAACAGGCTGAGTTTTGG		3		12	+1bp
		CCAACACAACTTCCTTATGATCACAAAAAAATGGGAGTTTCCCAGAAACAGGCTGAGTT		6			+4bp
		CCAACACAACTTCCTTATGATCACAAATGGGAGTTTCCCAGAAACAGGCTGAGTTTTGG	3				
	Embryo 3	CCAACACAACTTCCTTATGATCACAAATGGGAGTTTCACA------GGCTGAGTTTTGG			14	20	C>A, -6bp
		CCAACACAACTTCCTTATGATCACAAATGGGAGTTTCCCAGAAACAGGCTGAGTTTTGG	6				
		Total (%)	15 (33.3)	9 (20.0)	21 (46.7)	45	
nuclease	Embryo 1	CCAACACAACTTCCTTATGATCACAAAAAATGGGAGTTTCCCAGAAACAGGCTGAGTTT		7		13	+3bp
		CCACACAACTTCCTTA----------TGGGAGTTTCCCAGAAACAGGCTGAGTTTTGG			4		-10bp
		CCAACACAACTTCCTTATGATCACAAAATGGGAGTTTCCCAGAAACAGGCTGAGTTTTG		2			+1bp
	Embryo 2	CCAACACAACTTCCTTATGATCACA--TGGGAGTTTCCCAGAAACAGGCTGAGTTTTGG			3	15	-2bp
		CCAACACAACTTCCTTATGATCACAA-TGGGAGTTTCCCAGAAACAGGCTGAGTTTTGG			5		-1bp
		CCAACACAACTTCCTTATGATCACAAAAATGGGAGTTTCCCAGAAACAGGCTGAGTTTT		4			+2bp
		CCAACACAACTTCCTTATGATCACAAATGGGAGTTTCCCAGAAACAGGCTGAGTTTTGG	3				
	Embryo 3	CCAACACAACTTCCTTATGATCACAAAATGGGAGTTTCCCAGAAACAGGCTGAGTTTTG		11		21	+1bp
		CCAACACAACTTCCTTATGATCACAA-TGGGAGTTTCCCAGAAACAGGCTGAGTTTTGG			10		-1bp
		Total (%)	3 (6.1)	24 (49.0)	22 (44.9)	49	

Underlines indicated W37-1 sgRNA sequence. Bold letters showed E579 at c-*kit* gene exon11, and red letters indicated insertion/deletion modification of the target gene.
Ins; insertion modification, Del; deletion modification.

Supplementary Table 8. Sub-cloning analysis of CRISPR/Cas9-mediated *Shank3* gene modification in marmoset embryos

Injected materials	Embryo no.	Sequence	No. of sub-clones				Modified sequence
Intact sequence		CCCAGCAGGCTGGGG**GCC**GAAGAGGAGCGCCCGGGCACCCCGGAGTTGGCCCCGGCCCCC	Intact	Ins	Del	Total	
mRNA	Embryo 1	CCCAGCAGGCTGGGGGCCGAAGAGGAG------------------TTGGCCCCGGCCCCC			14	16	-18bp
		CCCAGCAGGCTGGGGGCCGAAGAGGAGCGCCCGGGCACCCCGGAGTTGGCCCCGGCCCCC	2				
	Embryo 2	CCCAGCAGGCTGGGGGC-------------------ACCCCGGAGTTGGCCCCGGCCCCC			9	12	-19bp
		CCCAGCAGGCTGGGGGCCGAAGAGGAG--CCCGGGCACCCCGGAGTTGGCCCCGGCCCCC			3		-2bp
	Embryo 3	CCC--------------------------------------GGAGTTGGCCCCGGCCCCC			8	15	-38bp
		CCCAGCAGGCTGGGGGCCGAAGAGGAGCGCCCGGGCACCCCGGAGTTGGCCCCGGCCCCC	7				
		Total (%)	9 (20.9)	0 (0)	34 (79.1)	43	
nuclease	Embryo 1	CCCAGCAGGCTGGG---------------------CACCCCGGAGTTGGCCCCGGCCCCC			9	19	-21bp
		CCCAGCAGGCTGGGGGCCG--------CGCCCGGGCACCCCGGAGTTGGCCCCGGCCCCC			10		-8bp
	Embryo 2	CCCAGCAGGCTGGGGGCCGAAGAGGAGCG----GGCACCCCGGAGTTGGCCCCGGCCCCC			8	13	-4bp
		CCGGTGTCTGCTCTGAAGCCATTGGTC-453del-TGAAGACCCCGGAGTTGGCCCCGGC			3*		-453bp, +5bp
		CCCAGCAGGCTGGGGGCCGAAGAGGAGCGCCCGGGCACCCCGGAGTTGGCCCCGGCCCCC	2				
	Embryo 3	CCCAGCAGGC--------------------------ACCCCGGAGTTGGCCCCGGCCCCC			18	18	-26bp
		Total (%)	2 (4.0)	0 (0)	48 (96.0)	45	

Underlines indicated Shk-A sgRNA sequence. Bold letters showed *Shank3* gene 1227 alanine, and red letters indicated insertion/deletion modification of the target gene.

Ins; insertion modification, Del; deletion modification.

* Modified sequence of large deletion containing small insertions was classified as "deletion" mutations in this table.

Supplementary Table 9. Mosaicism estimation of CRISPR/Cas9 injected embryos by blastomere analysis

Target	Injected materials	Embryo	No. of blastomere	No. of blastomere (%)			% of average of modified blastomere	% of completely modified embryo
				Intact	Modified			
					Bi-allele	Mono-allele		
c-*kit*	mRNA	1	10	6 (60)	2 (20)	2 (20)		
		2	10	3 (30)	4 (40)	3 (30)		
		3	8	6 (75)	2 (25)	0 (0)	50.0	0
		4	8	2 (25)	5 (62.5)	1 (12.5)		
		5	5	3 (60)	2 (40)	0 (0)		
		Total	41	20 (48.8)	15 (36.6)	6 (14.6)		
	nuclease	1	12	3 (25)	9 (75)	0 (0)		
		2	8	0 (0)	8 (100)	0 (0)		
		3	7	4 (57.1)	3 (42.9)	0 (0)	83.6	60
		4	7	0 (0)	7 (100)	0 (0)		
		5	6	0 (0)	6 (100)	0 (0)		
		Total	40	7 (17.5)	33 (82.5)	0 (0)		
Shank3	mRNA	1	9	3 (33.3)	6 (66.7)	0 (0)		
		2	8	2 (25)	5 (62.5)	1 (12.5)		
		3	8	8 (100)	0 (0)	0 (0)	56.3	20
		4	4	2 (50)	2 (50)	0 (0)		
		5	4	0 (0)	2 (50)	2 (50)		
		Total	33	15 (45.5)	15 (45.5)	1 (9.1)		
	nuclease	1	8	0 (0)	8 (100)	0 (0)		
		2	8	4 (50)	4 (50)	0 (0)		
		3	6	0 (0)	6 (100)	0 (0)	90.0	80
		4	5	0 (0)	5 (100)	0 (0)		
		5	5	0 (0)	5 (100)	0 (0)		
		Total	32	4 (12.5)	28 (87.5)	0 (0)		

Supplementary Table 10. Blastomeres analysis of CRISPR/Cas9 injected embryos targeting c-*kit* gene

Injected materials	Embryo no.	Sequence	No. of blastomere			Modified sequence
			Intact	Modified	Total	
Intact sequence		CCCAACACAACTTCCTTATGATCACAAATGG**GAG**TTTCCCAGAAACAGGCTGAGTTTTGG				
mRNA	Embryo 1	CCCAACACAACTTCCTTATGATCACAAATGGGAGTTTCCCAGAAACAGGCTGAGTTTTGG		2	10	Intact / -17bp
		CCCAACACAACTTCCTGTTTCCCACAAA-----------------CAGGCTGAGTTTTGG				
		CCCAACACAACTTCCTTATGATCACAAATGGGAGTTTCCCAGAAAACAGGCTGAGTTTTG		2		+1bp
		CCCAACACAACTTCCTTATGATCACAAATGGGAGTTTCCCAGAAACAGGCTGAGTTTTGG	6			Intact
	Embryo 2	CCCAACACAACTTCCTTATGATCACAAATGGGAGTTTCCCAGAAACAGGCTGAGTTTTGG		4	10	-12bp / +1bp
		CCCAACACAACTTCCTTATGAT-----------ATTTCCCAGAAACAGGCTGAGTTTTGG				
		CCCAACACAACTTCCTTATGATCACAAATGGGAGTTTCCCAGAAACAGGCTGAGTTTTGG		3		Intact / -2bp
		CCCAACACAACTTCCTTATGATCACA--TGGGAGTTTCCCAGAAACAGGCTGAGTTTTGG				
		CCCAACACAACTTCCTTATGATCACAAATGGGAGTTTCCCAGAAACAGGCTGAGTTTTGG	3			Intact
	Embryo 3	CCCAGACAAAAGCT----------------------------------GGCTGATTTTTGG		2	8	-37bp, +4bp, G>T/ -41bp, 4bp
		CCCAGAAACA--------------------------------------GACAGAGTTTGG				
		CCCAACACAACTTCCTTATGATCACAAATGGGAGTTTCCCAGAAACAGGCTGAGTTTTGG	6			Intact
	Embryo 4	CCCAACACAACTTCCT-----------------GTTTCCCAGAAACAGGCTGAGTTTTGG		4	8	-17bp
		CCCAACACAACTTCCT-----------------GTTTCCCAGAAACAGGCTGAGTTTTGG		1		Intact / -17bp
		CCCAACACAACTTCCTTATGATCACAAATGGGAGTTTCCCAGAAACAGGCTGAGTTTTGG				
		CCCAACACAACTTCCTTATGATCACAGGAAACAGGAAGTTTCCCAGAAACAGGCTGAGTT		1		-6bp, +10bp
		CCCAACACAACTTCCTTATGATCACAAATGGGAGTTTCCCAGAAACAGGCTGAGTTTTGG	2			Intact
	Embryo 5	CCCAACACAACTTCCTTATGATCATTATGAAATGGGAGTTTCCCAGAAACAGGCTGAGTT		2	5	-1bp, +5bp
		CCCAACACAACTTCCTTATGATCACAAATGGGAGTTTCCCAGAAACAGGCTGAGTTTTGG	3			Intact
nuclease	Embryo 1	CCCAACACAACTTCCTTATGATCACA--TGGGAGTTTCCCAGAAACAGGCTGAGTTTTGG		6	12	-2bp
		CCCAACACAACTTCCTTATG----------GGAGTTTCCCAGAAACAGGCTGAGTTTTGG		3		-10bp
		CCCAACACAACTTCCTTATGATCACAAATGGGAGTTTCCCAGAAACAGGCTGAGTTTTGG	3			Intact
	Embryo 2	CCCAACACAACTTCCTTATGATCACATAATCATAAGGGATGTTTAAGTATGATACCAATC AAAATGGGAGTTTCCCAGAAACAGGCTGAGTTTTGG		4	8	+36bp
		CCCAACACAACTTCCTTATGATCACA-ATGGGAGTTTCCCAGAAACAGGCTGAGTTTTGG		4		-1bp / +36bp
		CCCAACACAACTTCCTTATGATCACATAATCATAAGGGATGTTTAAGTATGATACCAATC AAAATGGGAGTTTCCCAGAAACAGGCTGAGTTTTGG				
	Embryo 3*	CCCAACACAACTTCCTTATGATCACAAATGGGAGTTTCA------CAGGCTGAGTTTTGG		3	7	-6bp
		CCCAACACAACTTCCTTATGATCACAAATGGGAGTTTCCCAGAAACAGGCTGAGTTTTGG	4			Intact
	Embryo 4	CCCAACACAACTTCCTTATGATCACAAAAATGGGAGTTTCCCAGAAACAGGCTGAGTTTT		4	7	+2bp / -2bp
		CCCAACACAACTTCCTTATGATCA-AATGGGAGTTTCCCAGAAACAGGCTGAGTTTTGG				
		CCCAACACAACTTCCTTATGATCACAAAAAATGGGAGTTTCCCAGAAACAGGCTGAGTTT		3		+3bp
	Embryo 5	CCCAACACAACTTCCTTATGATCACAAAAATGGGAGTTTCCCAGAAACAGGCTGAGTTTT		4	6	+2bp
		CCCAACACAACTTCCTTATGATCACAA-TGGGAGTTTCCCAGAAACAGG CTGAGTTTTGG		2		-1bp

Underlines indicated W37-1 sgRNA sequence. Bold letters showed E579 at c-*kit* gene exon11, and red letters indicated insertion/deletion modification of the target gene. Heterozygotic mutation sequences were filled by gray. *Correlate to Figure 3B.

Supplementary Table 11. Blastomeres analysis of CRISPR/Cas9 injected embryos targeting *Shank3* gene

Injected materials	Embryo no.	Sequence	No. of blastomere			Modified sequence
			Intact	Modified	Total	
Intact sequence		CCCAGCAGGCTGGGG**GCC**GAAGAGGAGCGCCCGGGCACCCCGGAGTTGGCCCCGGCCCCC				
mRNA	Embryo 1	CCCAGCAGGCTGGGGGCCGAAGAGGAGC----GGGCACCCCGGAGTTGGCCCCGGCCCCC		4	9	-4bp
		CCCAGCAGGCTGGGGGCCGAAGAGGAGC-CCCGGGCACCCCGGAGTTGGCCCCGGCCCCC		2		-1bp
		CCCAGCAGGCTGGGGGCCGAAGAGGAGCGCCCGGGCACCCCGGAGTTGGCCCCGGCCCCC	3			Intact
	Embryo 2	CCCAGCAGGCTGGGGGCCGAAGAGG----------CACCCCGGAGTTGGCCCCGGCCCCC		3	8	-10bp
		CCCAGCAGGCTGGGGGCCGAAGAGGAG--CCCGGGCACCCCGGAGTTGGCCCCGGCCCCC		2		-2bp
		CCCAGCAGGCTGGGGGCCGAAGAGGAGCGCCCGGGCACCCCGGAGTTGGCCCCGGCCCCC<br>CCCAGCAGGCTGGGGGC-------------------ACCCCGGAGTTGGCCCCGGCCCCC		1		Intact / -19bp
		CCCAGCAGGCTGGGGGCCGAAGAGGAGCGCCCGGGCACCCCGGAGTTGGCCCCGGCCCCC	2			Intact
	Embryo 3	CCCAGCAGGCTGGGGGCCGAAGAGGAGCGCCCGGGCACCCCGGAGTTGGCCCCGGCCCCC	8		8	Intact
	Embryo 4	CCCAGCAGGCTGGGGGCCGAAGAGGAGC----------CCCGGAGTTGGGTCCGAAGAGT<br>TGGCCCCGGCCCCCA		2	4	-10bp, +14bp
		CCCAGCAGGCTGGGGGCCGAAGAGGAGCGCCCGGGCACCCCGGAGTTGGCCCCGGCCCCC	2			Intact
	Embryo 5	CCCAGCAGGCTGGGGGCCGAAGAGGAGCGCCCGGGCACCCCGGAGTTGGCCCCGGCCCCC<br>CCCAGCAGGCTGGGG-CCGAAGAGG----------CACCCCGGAGTTGGCCCCGGCCCCC		2	4	Intact / -10bp
		CCCATCAGGCTGGGGGCCGAAGAGG----------CACCCCGGAGTTGGCCCCGGCCCCC		2		-10bp
nuclease	Embryo 1	CCCAGCAGGCTGGGGGCCGAAGAGGAGC-144bp del-TGGTGTTTGCTGT		5	8	-144bp
		CCCAGCAGGCTGGGGGCCGAAGAGGAGCCCCACCGCCGGCCGCGACTTGCTGCTGCCCTC<br>CCCGGTGTCTGCTCTGAAGCCATTGGTCTGAAGACCCCGGAGTTGGCCCCGGCCCCC		2		-8bp, +65bp
		CCCAGCAGGCTGGGGGCCGAAGAGGAG--CCCGGGCACCCCGGAGTTGGCCCCGGCCCCC		1		-2bp
	Embryo 2	CCCAGCAGGCTGGGGGCCGAAGAGGAG------------------TTGGCCCCGGCCCCC<br>CCCAGCAGGCTGGGGGCCGAAGAGGAGCGCCCGGGCACCCCGATGTAGGCAGCGCCCCC		4	8	-18bp / 13bp replacement
		CCCAGCAGGCTGGGGGCCGAAGAGGAGCGCCCGGGCACCCCGGAGTTGGCCCCGGCCCCC	4			Intact
	Embryo 3	CCCAGCAGGCTGGGGGCCGAAGAGGAGCG-----------------GGGCCCCGGCCCCC		6	6	-18bp, +1bp replacement
	Embryo 4	AGCAAC---<br>CCCGGAGTTGGCCCCGGCCCCC		5	5	-47bp
	Embryo 5	CCCAGCAGGCTGGGGGCCGAAGAGGAG------------------TTGGCCCCGGCCCCC		5	5	-18bp

Underlines indicated Shk-A sgRNA sequence. Bold letters showed *Shank3* gene 1227 alanine, and red letters indicated insertion/deletion modification of the target gene. Heterozygotic mutation sequences were filled by gray. Ins; insertion modification, Del; deletion modification.

Supplementary Table 12. Sequence patterns of the target gene after CRISPR/mRNA or CRISPR/nuclease injection into embryos

Target gene	Injected materials	No. of analyzed embryos	No. of the target gene modified embryos	No. of sequence patterns
c-*kit*	mRNA	5	5	3<br>3<br>2<br>4<br>2
Shank3		5	4	3<br>4<br>2<br>Intact<br>2
c-*kit*	nuclease	5	5	3<br>2<br>2<br>2<br>2
Shank3		5	5	3<br>2<br>1<br>1<br>1

Supplementary Table 13. Knock-in donor oligonucleotides sequence for c-*kit* gene

Donor ssODN	Sequence
36nt-S	5′-CTTATGATCATAAGTGGAAGTTTCCCAGAAACAGGC-3′
36nt-AS	3′-GAATACTAGTATTCACCTTCAAAGGGTCTTTGTCCG-5′
100nt-S	5′-ATAATTATGTTTACATAGACCCAACACAACTTCCTTATGATCATAAGTGGAAGTTTCCCAGAAACAGGCTGAGTTTTGGTCAGTATGAATTCGAAACAGG-3′
100nt-AS	3′-TATTAATACAAATGTATCTGGGTTGTGTTGAAGGAATACTAGTATTCACCTTCAAAGGGTCTTTGTCCGACTCAAAACCAGTCATACTTAAGCTTTGTCC-5′

Blue letters indicated the silent mutation to avoid the re-targeting by CRISRP/Cas9. Red letters indicate the point mutations to cause the amino acid substitutions in c-*kit* gene. Underlines indicated E579K mutation of c-*kit* gene exon 11.

Supplementary Table 14. Knock-in efficiency for c-*kit* gene in blastomere

Injected materials	ssODN	Embryo	No. of blastomere	No. of blastomere (%)				% of modified blastomere
				Intact	Modified			
					Indel	precise KI	imprecise KI	
mRNA	36nt-S	1	12	6 (50)	6 (50)	0 (0)	0 (0)	50
		2	12	3 (25)	9 (75)	0 (0)	0 (0)	75
		3	9	0 (0)	9 (100)	0 (0)	0 (0)	100
		4	8	2 (25)	6 (75)	0 (0)	0 (0)	75
		5	8	6 (75)	2 (25)	0 (0)	0 (0)	25
		Total	49	17 (34.7)	32 (65.3)	0 (0)	0 (0)	
	36nt-AS	1	9	2 (22.2)	7 (77.8)	0 (0)	0 (0)	77.8
		2	8	3 (37.5)	5 (62.5)	0 (0)	0 (0)	62.5
		3	8	0 (0)	8 (100)	0 (0)	0 (0)	100
		4	7	2 (28.6)	4 (57.1)	0 (0)	1 (14.3) *	71.4
		5	5	4 (80)	1 (20)	0 (0)	0 (0)	20
		Total	37	11 (29.7)	25 (67.6)	0 (0)	1 (2.7)	
	100nt-S	1	8	3 (37.5)	5 (62.5)	0 (0)	0 (0)	62.5
		2	8	3 (37.5)	5 (62.5)	0 (0)	0 (0)	62.5
		3	7	1 (14.3)	6 (85.7)	0 (0)	0 (0)	85.7
		4	7	5 (71.4)	2 (28.6)	0 (0)	0 (0)	28.6
		5	6	3 (50)	3 (50)	0 (0)	0 (0)	50
		Total	36	15 (41.7)	21 (58.3)	0 (0)	0 (0)	
	100nt-AS	1	8	0 (0)	8 (100)	0 (0)	0 (0)	100
		2	8	5 (62.5)	3 (37.5)	0 (0)	0 (0)	37.5
		3	8	6 (75)	2 (25)	0 (0)	0 (0)	25
		4	8	7 (87.5)	1 (12.5)	0 (0)	0 (0)	12.5
		5	7	1 (14.3)	6 (85.7)	0 (0)	0 (0)	85.7
		Total	39	19 (48.7)	20 (51.3)	0 (0)	0 (0)	
nuclease	36nt-S	1	11	0 (0)	9 (81.8)	0 (0)	2 (18.1)	100
		2	8	0 (0)	4 (50)	4 (50)	0 (0)	100
		3	7	0 (0)	4 (57.1)	3 (42.9) *	0 (0)	100
		4	6	0 (0)	3 (50)	3 (50)	0 (0)	100
		5	6	0 (0)	4 (66.7)	2 (33.3) *	0 (0)	100
		Total	38	0 (0)	24 (63.2)	12 (31.6)	2 (5.3)	
	36nt-AS	1	11	5 (45.5)	1 (9.1)	0 (0)	5 (45.5) *	54.5
		2	9	0 (0)	3 (33.3)	0 (0)	6 (66.7) *	100
		3	8	0 (0)	2 (25)	0 (0)	6 (75) *	100
		4	8	0 (0)	8 (100)	0 (0)	0 (0)	100
		5	6	0 (0)	3 (50)	3 (50)	0 (0)	100
		Total	42	5 (11.9)	17 (40.5)	3 (7.1)	17 (40.5)	
	100nt-S	1	10	10 (100)	0 (0)	0 (0)	0 (0)	0
		2	8	7 (87.5)	1 (12.5)	0 (0)	0 (0)	12.5

	3	8	7 (87.5)	1 (12.5)	0 (0)	0 (0)	12.5
	4	7	7 (100)	0 (0)	0 (0)	0 (0)	0
	5	6	6 (100)	0 (0)	0 (0)	0 (0)	0
	Total	39	37 (94.9)	2 (5.1)	0 (0)	0 (0)	
100nt-AS	1	8	7 (87.5)	1 (12.5)	0 (0)	0 (0)	12.5
	2	8	8 (100)	0 (0)	0 (0)	0 (0)	0
	3	8	8 (100)	0 (0)	0 (0)	0 (0)	0
	4	6	4 (66.7)	2(33.3)	0 (0)	0 (0)	33.3
	5	4	2 (50)	2 (50)	0 (0)	0 (0)	50
	Total	34	29 (85.3)	5 (14.7)	0 (0)	0 (0)	

*Included the number of heterozygous mutations.

Supplementary Table 15. Sequence results of c-*kit* gene Knock-in targeting

Injected materials	ssODN	Embryo no.	Sequence	No. of blastomere			Modified sequence
				Intact	Modified	Total	
	Intact sequence		CCCAACACAACTTCCTTATGATCACAAATGG**GAG**TTTCCCAGAAACAGGCTGAGTTTTGG				
	KI sequence		CCCAACACAACTTCCTTATGATCATAAGTGGAAGTTTCCCAGAAACAGGCTGAGTTTTGG				
mRNA	36nt-S	Embryo 1	CCCAACACAACTTCCTTATGATCACAAATGGGAGTTTCCCAGAAACAGGCTGAATTTTGG<br>CCCAACACAACTTCCTTATGATCACAAAATGGGAGTTTCCCAGAAACAGGCTGGGTTTTGG		4	12	Intact / +1bp
			CCCAACACAACTTCCTTATGATCACAAATGGGAGTTTCCCAGAAAACAGGCTGAGTTTTGG		2		+1bp
			CCCAACACAACTTCCTTATGATCACAAATGGGAGTTTCCCAGAAACAGGCTGAGTTTTGG	6			Intact
		Embryo 2	CCCAACACAACTTCCTTATGATCACAAATGGGAGTTTCCCAGAAACAGGCTGAGTTTTG<br>CC-AACACAACTTCCTTATGATCACAAAATGGGAGTTTCCCAGAAACAGGCT		3	12	Intact / +1bp
			CCCAACACAACTTCCTTATGATCACAAATGGGAGTTTCCCAGAAACAGGCTGAGTTTTGG<br>CCCAACACAACTTCCTTATGATCACAAA-----GTTTCCCAGAAACAGGCTGAGTTTTGG		4		Intact / -5bp
			CCCAACACAACTTCCTTATGATCACAAAATGGGAGTTTCCCAGAAACAGGCTGAGTTTTGG		1		+1bp
			CCCAACACAACTTCCTTATGATCACAAAAAATGGGAGTTTCCCAGAAACAGGCTGAGTTTT		1		+3bp
			CCCAACACAACTTCCTTATGATCACAAATGGGAGTTTCCCAGAAACAGGCTGAGTTTTGG	3			Intact
		Embryo 3	CCCAACACAACTTCCTTATGATCACA--TGGGAGTTTCCCAGAAACAGGCTGAGTTTTGG		4	9	-2bp
			CCCAACACAACTTCCTTATGATCACAAAATGGGAGTTTCCCAGAAACAGGCTGAGTTTTGG		3		+1bp
			CCCAACACAACTTCCTTATGATCACAA-TGGGAGTTTCCCAGAAACAGGCTGAGTTTTGG		2		-1bp
		Embryo 4	CCCAACACAACTTCCTTGTGATCACATACAAAGCTTAAGTATGACTGGTTTTGAGTCGGAC<br>AAAGACTCTGTTTCCCAGAAACAGGCTGAGTTTTGG		5	8	-7bp, +44bp
			CCCAACACAACTTCCTTATGATCACATAATCATAAGGGATGTTTAAGTATGATACCAATCA<br>AAATGGGAGTTTCCCAGAAACAGGCTGAGTTTTGG		1		+36bp
			CCCAACACAACTTCCTTATGATCACAAATGGGAGTTTCCCAGAAACAGGCTGAGTTTTGG	2			Intact
		Embryo 5	CCCAACACAACTTCCTTATGAT---TTCTGGGAGTTTCCCAGAAACAGGCTGAGTTTTGG		2	8	-6bp, +3bp
			CCCAACACAACTTCCTTATGATCACAAATGGGAGTTTCCCAGAAACAGGCTGAGTTTTGG	6			Intact
	36nt-AS	Embryo 1	CCCAACACAACTTCCTTATGATCA--AATGGGAGTTTCCCAGAAACAGGCTGAGTTTTGG<br>CCCAACACAACTTCCTTATGATCACAAATGGGAGTTTCCCAGAAACAGGCTGAGTTTTGG		5	9	Intact / -2bp
			CCCAACACAACTTCCTTATGATCACAAAAAAAATGGGAGTTTCCCAGAAACAGGCTGAGT		2		+5bp
			CCCAACACAACTTCCTTATGATCACAAATGGGAGTTTCCCAGAAACAGGCTGAGTTTTGG	2			Intact
		Embryo 2	CCCAACACAACTTCCTTATGATCAC--ATGGGAGTTTCCCAGAAACAGGCTGAGTTTTG<br>CCCAACAACAACTTCCTTATGATCACAAAATGGGAGTTTCCCAGAAACAGGCTGAGTTTT		3	8	-2bp / C>A, G>A
			CCCAACACAACTTCCTTATGATCACAAATGGGAGTTTCCCAGAAACAGGCTGAGTTTTGG<br>CC-AACACAATCTCCTTATGATCACATTATGGGAGTTTCCCAGAAACAGGCTGAGTTTTG		2		Intact / CT>TC, AA>TT
			CCCAACACAACTTCCTTATGATCACAAATGGGAGTTTCCCAGAAACAGGCTGAGTTTTGG	3			Intact
		Embryo 3	CCCAACACAACTTCCTTATGATCACAAATTTGGAAGTTTCCCAGAAACAGGCTGAGTTTT<br>CCTTGAAGTTCTCCAGAAACAGGCTGTTAAGTTTGCTGAGTATAAGACGGAGGTTTTGCA<br>TTTCACAGTA		6	8	GG>TT, G>A / large replacement
			CCCAACA--------------------------GTTTCCCAGAAACAGGCTGAGTTTTGG		2		-26bp
		Embryo 4	CCCAACACAACTTCCTTATGATCACAAATGGGAGTTTCCCACAAACAGGCTGAGTTTTG		1	7	G>C / imprecise KI

			CAACACAACTTCCTTATGATCACATAAGTGGAAGTTTCCGAAAAACAGGCTTAGTTTTGG				
			CACAACTTCCTTATGATCACAAAAATGGGAGTTTCCCAGAAACAGGCTGAGTTTTGG		4		+2bp
			CCCAACACAACTTCCTTATGATCACAAATGGGAGTTTCCCAGAAACAGGCTGAGTTTTGG	2			Intact
		Embryo 5	CCCAACACAACTTCCTTATGATCACAAATGGGAGTTTCCCAGAAACAGGCTGAGTTTTG<br>CCCAACACAACTTCCTTATGATCACAAAATGGGAGTTTCCCAGAAACAGGCTGGATTTTG		1	5	Intact / +1bp, AG>GA
			CCCAACACAACTTCCTTATGATCACAAATGGGAGTTTCCCAGAAACAGGCTGAGTTTTGG	4			Intact
	100nt-S	Embryo 1	CCCAACACAACTTCCTTATGATCACAAATGGGAGTTTCCCAGAAACAGGCTGAATTTTGG<br>CCCAACACAACTTCCTTATGATCACAAAAAATGGGAGTTTCCCAGAAACAGGCTGAATTT		3	8	Intact / +3bp
			CCCAACACAACTTCCTTATGATCACAAAAAAATGGGAGTTTCCCAGAAACAGGCTGAGTT		2		+4bp
			CCCAACACAACTTCCTTATGATCACAAATGGGAGTTTCCCAGAAACAGGCTGAGTTTTGG	3			Intact
		Embryo 2	CCCAACACAACTTCCTTATG----TTTCTGGGAGTTTCCCAGAAACAGGCTGAGTTTTGG		5	8	-8bp, +4bp
			CCCAACACAACTTCCTTATGATCACAAATGGGAGTTTCCCAGAAACAGGCTGAGTTTTGG	3			Intact
		Embryo 3	CCCAACACAACTTCCTTATGATCACAAAAATGGGAGTTTCCCAGAAACAGGCTGAGTTTT<br>CCCAACACAACTTCCTTATGATCACAAATGGGAGTTTCCCAGAAACAGGCTGAGTTTTG		3	7	Intact / +2bp
			CCCAACACAACTTCCTTATGATCACAAAAAAAATGGGAGTTTCCCACAAACACGCGGAGTT<br>CCCAACACAACTTCCTTATGATCACAAAATGGGAGTTTCCCACAAACACGCGGAGTTTTGG		3		+4bp / +1bp
			CCCAACACAACTTCCTTATGATCACAAATGGGAGTTTCCCAGAAACAGGCTGAGTTTTGG	1			Intact
		Embryo 4	CCCAACACAACTTCCTTATGATCACATGGGATCACAATGGGAGTTTCCCAGAAACAGGCTG		2	7	+9bp
			CCCAACACAACTTCCTTATGATCACAAATGGGAGTTTCCCAGAAACAGGCTGAGTTTTGG	5			Intact
		Embryo 5	CCCAACACAACTTCCTTATGATCACAAAATGGGAGTTTCCCAGAAACAGGCTGAGTTTTGG		2	6	+1bp
			CCCAACACAACTTCCTTATGATCACAAATGGGAGTTTCCCAGAAACAGGCTGAGTTTTGG<br>CCCAACACAACTTCCTTATGATCACAAAATGGGAGTTTCCCAAAAACAGGCTGAGTTTTGG		1		Intact / +1bp
			CCCAACACAACTTCCTTATGATCACAAATGGGAGTTTCCCAGAAACAGGCTGAGTTTTGG	3			Intact
	100nt-AS	Embryo 1	CCCAACACAACTTCCTTATG----------GGAGTTTCCCAGAAACAGGCTGAGTTTTGG<br>CCCAACACAACTTCCTTATG-----------GAGTTTCCCAGAAACAGGCTGA-TTTTTGG		3	8	-10bp / -11bp
			CCCAACACAACTTCCTTATGATCA---------GTTTCCCAGAAACAGGCTGAGTTTTGG<br>CCCAACACAACTTCCTTATGATCACAAAATGGGAGTTTCCCTGAATCTTGCTGAGTTTTGG		2		-9bp / +1bp
			CCCAACACAACTTCCTTATG----------GGAGTTTCCCAGAAACAGGCTGAGTTTTGG		2		-10bp
			CCCAACACAACTTCCTTATGATCACAAAATGGGAGTTTCCCAGAAACAGGCTGAGTTTTGG		1		1bp
		Embryo 2	CCCAACACAACTTCCTTATGA----AATGGGAGTTTCCCAGAAACAGGCTGAGTTTTGG		3	8	-4bp
			CCCAACACAACTTCCTTATGATCACAAATGGGAGTTTCCCAGAAACAGGCTGAGTTTTGG	5			Intact
		Embryo 3	CCCAACACAACTTCCTTATGATCACAAAATGGGAGTTTCCCAGAAACAGGCTGAGTTTTGG<br>CCCAACACAACTTCCTTATGATCACACAAATGGGAGTTTCCCAGAAACAGGCTGAGTTTTG		2	8	+1bp / +2bp
			CCCAACACAACTTCCTTATGATCACAAATGGGAGTTTCCCAGAAACAGGCTGAGTTTTGG	6			Intact
		Embryo 4	CCCAACACAACTTCCTGGTTGTGTTCC-TGGGAGTTTCCCAGAAACAGGCTGAGTTTTGG		1	8	-12bp, +11bp
			CCCAACACAACTTCCTTATGATCACAAATGGGAGTTTCCCAGAAACAGGCTGAGTTTTGG	7			Intact
		Embryo 5	CCCAACACAACTTCCTTATGATCACTCCCTTATGGGAGTTTCCCAGAAACAGGCTGAGTTT		4	7	-2bp, +6bp
			CCCAACACAACTTCCTTATGATCACA--TGGGAGTTTCCCAGAAACAGGCTGAGTTTTGG<br>CCCAACACAACTTCCTTATGATCAAAAAAATGGGAGTTTCCCAGAAACAGGCTGAGTTGTT<br>AGTCAGTAT		2		-2bp / +4bp,<br>-3bp, +4bp
			CCCAACACAACTTCCTTATGATCACAAATGGGAGTTTCCCAGAAACAGGCTGAGTTTTGG	1			Intact
nuclease	36nt-S	Embryo 1	CCCAACACAACTTCCTTATGATCACATAAGTGGAAGTTTCCCAGAAACAGGCTGAGTTTTG		2	11	imprecise KI

			CCCAACACAACTTCCTTATGATCACAAAATGGGAGTTTCCCAGAAACAGGCTGAGTTTTGG		4		+1bp
			CCCAACACAACTTCCTTATGATCA--AATGGGAGTTTCCCAGAAACAGGCTGAGTTTTGG		3		-2bp
			CCCAACACAACTTCCTTATGATCACAA-TGGGAGTTTCCCAGAAACAGGCTGAGTTTTGG		2		-1bp
		Embryo 2	CCCAACACAACTTCCTTATGATCATAAGTGGAAGTTTCCCAGAAACAGGCTGAGTTTTGG		4	8	KI
			CCCAACACAACTTCCTTATGATCACAAAATGGGAGTTTCCCAGAAACAGGCTGAGTTTTGG		4		+1bp
		Embryo 3	CCCAACACAACTTCCTTATGATCATAAGTGGAAGTTTCCCAGAAACAGGCTGAGTTTTGG		2	7	KI
			CCCAACACAACTTCCTTATGATCACAAATGGGAGTTTCCCAGAAACAGGCTGAATTTTGG<br>CCCAACACAACTTCCTTATGATCATAAGTGGAAGTTTCCCAGAAACAGGCTGAGTTTTG		1		Intact / KI
			CCCAACACAACTTCC--------CAGAATGGGAGTTTCCCAGAAACAGGCTGAGTTTTGG		4		-11bp, +3bp
		Embryo 4	CCCAACACAACTTCCTTATGATCATAAGTGGAAGTTTCCCAGAAACAGGCTGAGTTTTGG		3	6	KI
			CCCAACACAACTTCCTTATGATCACAAAATGGGAGTTTCCCAGAAACAGGCTGAGTTTTGG		3		+1bp
		Embryo 5	CCCAACACAACTTCCTTATGATCATAAGTGGAAGTTTCCCAGAAACAGGCTGAGTTTTGG<br>CCCAACACAACTTCCTTATGAT-----------GTTTCCCAGAAAGAGGTTGAGTTTTGG		2	6	-11bp / KI
			CCCAACACAACTTCCTTATGATCACAA-TGGGAGTTTCCCAGAAACAGGCTGAGTTTTGG		4		-1bp
	36nt-AS	Embryo 1	CCCAACACAACTTCCTTATGATCAC-------------CCAGAAACAGGCTGAGTTTTGG<br>CCCAACACAACTTCCTTATGATCACATAAGTGGAAGTTTCCCAGAAACAGGCTGAGTTTTG		5	11	-13bp / imprecise KI
			CCCAACACAACTTCCTTATGATCAC-------------CCAGAAACAGGCTGAGTTTTGG		1		-13bp
			CCCAACACAACTTCCTTATGATCACAAATGGGAGTTTCCCAGAAACAGGCTGAGTTTTGG	5			Intact
		Embryo 2	CCCAACACAACTTCCTTATGATCACAAATGGGAGTTTCCCAGAAACAGGCTGAGTTTTGG<br>CCCAACACAACTTCCTTATGATCACATAAGTGGAAGTTTCCCAGAAACAGGCTGAGTTTTG		4	9	Intact / imprecise KI
			CCCAACACAACTTCCTTATGATCACATAAGTGGAAGTTTCCCAGAAACAGGCTGAGTTTTG<br>CCCAACACAACTTCCTTATGATCACAAGTGGAAGTTTCCCAGAAACAGGCTGAGTTTTGG		2		A>G / imprecise KI
			CCCAACACAACTTCCTTATGATCACAAAATGGGAGTTTCCCAGAAACAGGCTGAGTTTTGG		3		+1bp
		Embryo 3	CCCAACACAACTTCCTTATGATCACAAATGGGAGTTTCCCAGAAACAGGCTGAGTTTTGG<br>CCCAACACAACTTCCTTATGATCATCATAAGTGGAAGTTTCCCAGAAACAGGCTGAGTTTT		6	8	Intact / imprecise KI
			CCCAACACAACTTCCTTATGATCACAAAAAAATGGGAGTTTCCCAGAAACAGGCTGAGTTT		2		+4bp
		Embryo 4	CCCAACACAACTTCCTTATG----------GGAGTTTCCCAGAAACAGGCTGAGTTTTGG		6	8	-10bp
			CCCAACACAACTTCCTTATGATCACAAAATGGGAGTTTCCCAGAAACAGGCTGAGTTTTGG		2		+1bp
		Embryo 5	CCCAACACAACTTCCTTATGATCATAAGTGGAAGTTTCCCAGAAACAGGCTGAGTTTTGG		3	6	KI
			CCCAACACAACTTCCTTATGATCACACAATGGGAGTTTCCCAGAAACAGGCTGAGTTTTGG		3		+1bp
	100nt-S	Embryo 1	CCCAACACAACTTCCTTATGATCACAAATGGGAGTTTCCCAGAAACAGGCTGAGTTTTGG	10		10	Intact
		Embryo 2	CCCAACACAACTTCCTTATGATCACAAATGGGAGTTTCCCAGAAACAGGCTGAATTTTGG<br>CCCAACACAACTTCCTTATGATCACAAAAAATGGGAGTTTCCCAGAAACAGGCTGAATTTT		1	8	Intact / +3bp
			CCCAACACAACTTCCTTATGATCACAAATGGGAGTTTCCCAGAAACAGGCTGAGTTTTGG	7			Intact
		Embryo 3	CCCAACACAACTTCCTTATGATCACAAAATGGGAGTTTCCCAGAAACAGGCTGAGTTTTGG		1	8	+1bp
			CCCAACACAACTTCCTTATGATCACAAATGGGAGTTTCCCAGAAACAGGCTGAGTTTTGG	7			Intact
		Embryo 4	CCCAACACAACTTCCTTATGATCACAAATGGGAGTTTCCCAGAAACAGGCTGAGTTTTGG	7		7	Intact
		Embryo 5	CCCAACACAACTTCCTTATGATCACAAATGGGAGTTTCCCAGAAACAGGCTGAGTTTTGG	6		6	Intact
	100nt-AS	Embryo 1	CCCACCACAACTTCCTTATGATCACAAATGGGAGTTTCCCAGAAACAGGCTGAGTTTTG<br>CCCAAAACAACTTCCTTATGATCACAAAATGGGAGTTTCCCAGAAACAGGCTGAGTTTTG		1	8	Intact / CC>AA, +1bp
			CCCAACACAACTTCCTTATGATCACAAATGGGAGTTTCCCAGAAACAGGCTGAGTTTTGG	7			Intact
		Embryo 2	CCCAACACAACTTCCTTATGATCACAAATGGGAGTTTCCCAGAAACAGGCTGAGTTTTGG	8		8	Intact

		Embryo 3	CCCAACACAACTTCCTTATGATCACAAATGGGAGTTTCCCAGAAACAGGCTGAGTTTTGG	8		8	Intact
		Embryo 4	CCCAACACAACTTCCTTATGAT-------GGGAGTTTCCCAGAAACAGGCTGAGTTTTG		2	6	-7bp
			CCCAACACAACTTCCTTATGATCACAAATGGGAGTTTCCCAGAAACAGGCTGAGTTTTGG	4			Intact
		Embryo 5	CCCAACACA------------------------GTTTCCCAGAAACAGGCTGAGTTTTG		1	4	-24bp
			CCCAACACAACTTCCTTATGATCACAAAATGGGAGTTTCCCAGAAACAGGCTGAGTTTTG		1		+1bp
			CCCAACACAACTTCCTTATGATCACAAATGGGAGTTTCCCAGAAACAGGCTGAGTTTTGG	2			Intact

Bold letters showed E579 at c-*kit* gene exon11 and underlines indicated W37-1 sgRNA sequence. Blue letters indicated KI mutation derived from donor ssODN, and red letters indicated insertion/deletion sequences.

Heterozygotic mutation sequences are filled by gray.

Supplementary Table 16. Knock-in experiments for c-*kit* gene in marmoset embryos

Injected materials	ssODN	Injected	Development			Analyzed	Modified (%) **	KI (%) **
			Dead	1-7cell	8-12cell (%) *			
nuclease	-	14	2	9	3 (21.4)	3	3 (100)	-
	36nt-S	32	5	11	16 (50)	13	12 (92.3)	4 (30.8)
	36nt-AS	29	7	7	15 (51.7)	12	12 (100)	1 (8.3)
	100nt-S	27	5	9	13 (48.1)	10	2 (20)	0 (0)
	100nt-AS	29	4	12	13 (44.8)	10	3 (30)	0 (0)

*Developmental stage of *in vitro* cultured embryos at Day5 after injection, and the developmental efficiencies were calculated from the number of injected embryos.

**Calculated from the number of analyzed embryos.

Supplementary Table 17. Off-targets

sgRNA	sgRNA sequence	Off-target sequence	No. of mismatch	Off-target Score*	Region	
W37-1	ACTTCCTTATGATCACAAATGGG	TCTTCATTATGATCACAAAAAGG	3	0.522321429	NC_013908.1	intergenic
		GATTCCTTTTGATCACAAATAGG	3	0.436363636	NC_013908.1	intergenic
		ACATCCTTAAGATCACAAAATGG	3	0.344387755	NC_013918.1	intergenic
		ACTTGCTTAAGATCACAAAATGG	3	0.289285714	NC_013897.1	intron
		ACTCTCTTATGTTCACAAATCGG	3	0.137254902	NC_013910.1	intergenic
		ACTTGGTTATTATCACAAATAGG	3	0.115384616	NC_013896.1	intergenic
		ACTTCCTTGTCATCACACATAGG	3	0.054421769	NC_013900.1	intergenic
		AATTCCCTATGATCACAAACTGG	3	0.045454545	NC_013897.1	intron
W37-2	AATGGGAGTTTCCCAGAAACAGG	AATGGGAGAAACCCAGAAACAGG	3	0.596938776	NW_003185663.1	intergenic
		TATGGGGGTTTCCCAGAATCAGG	3	0.380090497	NC_013903.1	intergenic
		AATGGGAATTCCCCAGAGACCGG	3	0.266666667	NC_013898.1	exon
		AGAGGGTGTTTCCCAGAAACGGG	3	0.25	NC_013907.1	intergenic
		AATGGGAGTAACCCAGAAGCAGG	3	0.241071429	NC_013905.1	intergenic
		AACAGGAGTTTTCCAGAAACAGG	3	0.207692308	NC_013909.1	intron
		AGTGGGAGGTTCCCAGAAGCTGG	3	0.185714286	NC_013918.1	intron
		AATGTGAGTTTCCCAAAAAAAGG	3	0.15	NC_013905.1	intergenic
		AATGGGTGTATCCCAGAAGCAGG	3	0.140625	NC_013909.1	intron
		CATGAGAGTTTCCCAGGAACCGG	3	0.131092437	NC_013896.1	intergenic
		ATTGGGAATTTCCCAGGAACGGG	3	0.128342246	NC_013918.1	exon
		AATGGGAGTTAGACAGAAACTGG	3	0.128205128	NC_013915.1	intergenic
		AATGGGAGTTTCTAAGATACTGG	3	0.1225	NC_013896.1	intergenic
		AATGGGATTTTCCAAGAAGCAGG	3	0.08203125	NC_013910.1	intergenic
		AATGTGAGTTTCACAGAATCGGG	3	0.062130178	NC_013907.1	exon
		AAGGGGAGTTTCCAAGAACCAGG	3	0.036206897	NC_013902.1	intergenic
		AGTGGGAGTTTCCCAGGCACTGG	3	0.026890756	NC_013915.1	intergenic
		AAGGGGAGTTTCCCTGCAACAGG	3	0.017647059	NC_013912.1	intergenic
W37-3	AACTTCCTTATGATCACAAATGG	ATCTTCTTTATGATCACATAAGG	3	0.318181818	NC_013911.1	intergenic
		AACATCATTATGGTCACAAATGG	3	0.232919255	NC_013916.1	intergenic
		ATCTCCCTTATGACCACAAATGG	3	0.207792208	NC_013901.1	intron
		AACTTCCCTATGGTCACACAGGG	3	0.134932534	NW_003188020.1	intron
		AACTGTCTTATGATAACAAAAGG	3	0.132063492	NC_013908.1	intergenic
		AACTTTCTGATGATCACCAAAGG	3	0.109491415	NC_013901.1	intron
		AACTTCCTTAATTTCACAAAGGG	3	0.086538462	NC_013906.1	intergenic
		AACTTCTTTATTATCACACAAGG	3	0.064655173	NC_013907.1	intergenic
		AACCTCCTTATGATCAGAATAGG	3	0.02283737	NC_013897.1	intron
		AACTTTGTTATGATGACAAAAGG	3	0.021848739	NC_013900.1	intergenic
		AACTTCTTTATTATCAGAAAAGG	3	0.018382353	NC_013908.1	intergenic
		AACTTCCTTAAGATGAGAAAGGG	3	0.002205882	NC_013912.1	intergenic
W37-4	GATCATAAGGAAGTTGTGTTGGG	AATTATAAAGAAGTTGTGTTAGG	3	0.462857143	NC_013911.1	intron
		AATCATAAAGAAGTTGTATTTGG	3	0.40054945	NC_013907.1	intergenic

		GACCAAAAGGAAATTGTGTTTGG	3	0.342857143	NC_013896.1	intergenic
		GACCATAGGAAAGTTGTGTTGGG	3	0.293333333	NC_013902.1	intergenic
		GCTCAGAAGCAAGTTGTGTTGGG	3	0.179591837	NC_013897.1	intergenic
		GATAATAAAGAAGTTGTGATTGG	3	0.154672395	NC_013907.1	intergenic
		GATCAAATGGAAGTTGTGCTGGG	3	0.102463054	NC_013896.1	intron
		AATCTTAAGGAAGTTGTGATAGG	3	0.093506494	NC_013904.1	intron
		GGTCATAAGGAAGATGTGTGTGG	3	0.087394958	NC_013900.1	intron
		GATGAGAAGGAAGTTGTGGTTGG	3	0.071428571	NC_013900.1	exon
		GATCATAAAGAAGAGGTGTTTGG	3	0.019897959	NC_013898.1	intergenic
		GATCATTAAGAAGTGGTGTTGGG	3	0.0140625	NC_013918.1	intergenic
Shk-A	GGGGCCGAAGAGGAGCGCCCGGG	GGGGCCAAAGAGGAGAGCCAAGG	3	0.5	NC_013896.1	intergenic
		GAGGCCGAAAAGGTGCGCCCGGG	3	0.421196581	NC_013900.1	intergenic
		GGGGCCGGAGAGGTGAGCCCGGG	3	0.391111111	NC_013901.1	intergenic
		GGGGCAGAAGAGCAGAGCCCTGG	3	0.390977444	NC_013900.1	intron
		AGGGCCGCAGAGGAGAGCCCAGG	3	0.385714286	NC_013900.1	intergenic
		GGGGCTGAAGAAGAGCCCCCAGG	3	0.203921569	NC_013901.1	intergenic
		GGAGCAGAAGAGGAGCCCCCAGG	3	0.163865547	NC_013896.1	intron
		GGGGCGGAAGGGGAGTGCCCTGG	3	0.1	NC_013902.1	intergenic
		GGGGCAGATGAGGAGGGCCCGGG	3	0.085714286	NC_013906.1	intergenic
		GGGGCCAAAGAGGAGTTCCCTGG	3	0.076923077	NC_013914.1	intergenic
		GGGGAGGAAGAGGAGGGCCCTGG	3	0.043956044	NC_013909.1	intergenic
		GGGGCCGAGGAGGCGCGCCCGGG	2	0.142857143	NC_013896.1	intergenic
Shk-B	GGGCTCCTGCCCGTTGCTGGTGG	GGGTCACTGCCCGTTGCTGGTGG	3	0.742857143	NC_013900.1	intergenic
		GGACTTCTGCCCATTGCTGGAGG	3	0.642857143	NC_013902.1	intron
		AGGCTCCAGCCTGTTGCTGGTGG	3	0.387692307	NC_013918.1	intron
		GGGCTCCGGCCCGTTGCATGTGG	3	0.325925926	NW_003192188.1	intergenic
		GGGCTACTGCAGGTTGCTGGGGG	3	0.309523809	NC_013918.1	intergenic
		GGGCTCCAGCCTGTTGCTGTGGG	3	0.301538461	NC_013898.1	intergenic
		GGGCTCCTCCCTGTTGCTGAGGG	3	0.271819526	NC_013902.1	intergenic
		GGGCTCCTCCCTGTTGCTGTGGG	3	0.20295858	NW_003187796.1	intergenic
		GGGCTACTGCCATTTGCTGGGGG	3	0.198979592	NC_013918.1	intergenic
		CGGCTCCTGCCCACTGCTGGAGG	3	0.188383046	NC_013900.1	intergenic
		GGGCTCCTGTCTGCTGCTGGCGG	3	0.14479638	NC_013907.1	intron
		GGTGTGCTGCCCGTTGCTGGGGG	3	0.125	NW_003188042.1	intron
		GGGCTCTTGCTGGTTGCTGGTGG	3	0.111111111	NC_013899.1	intergenic
		GGGCTGCTGCCATTTGCTGGGGG	3	0.107142857	NC_013913.1	intron
		GGGCTCCTCCCTGCTGCTGGGGG	3	0.082840237	NC_013914.1	intergenic
		GGGCTGCTGCCGTTTGCTGGGGG	3	0.066666667	NC_013902.1	intergenic
		GGGCTGCTGCCAGTTGGTGGAGG	3	0.021008403	NC_013900.1	intergenic
Shk-C	AGAGGAGCGCCCGGGCACCCCGG	AGAGCAGCACCCAGGCACCCAGG	3	0.466248038	NC_013897.1	intergenic
		AGAGGAGGCCCCAGGCACCCAGG	3	0.319526627	NC_013902.1	intron
		AGAGGGGCTCCAGGGCACCCGGG	3	0.272108843	NC_013900.1	intergenic
		GGAAGAGCGCCCGGGCACCTCGG	3	0.27	NC_013906.1	intergenic

AGAGCAGCCCCCGGGCACACAGG	3	0.181318681	NC_013896.1	intergenic
AGAGGAGCCGACGGGCACCCGGG	3	0.157051282	NC_013906.1	intergenic
AGAGCAGCACCCTGGCACCCAGG	3	0.151530612	NC_013904.1	intergenic
TGAGGAGCGCCCGGAGACCCAGG	3	0.14479638	NC_013913.1	exon
CGAGGAACGCCCGGGGACCCAGG	3	0.131868132	NC_013906.1	intergenic
AGAGCAGCGCCCAGGCGCCCAGG	3	0.127989657	NC_013917.1	exon
CGAAGAGCGCCCGGGGACCCAGG	3	0.118681319	NC_013901.1	intergenic
GGAGGAGCGCCCGCGCGCCCGGG	3	0.075630252	NC_013896.1	exon
AGAGGAGGGCCCGGGCTCTCTGG	3	0.03956044	NC_013901.1	intron
AGAGAAGCGCCCTGGCACCGTGG	3	0.015294118	NC_013896.1	intron
AGAGGTGCGCCCGGGCAGGCTGG	3	0.011904762	NC_013896.1	intergenic
AGAGGAGCGGCCGGGCGCCGTGG	3	0.004036909	NC_013917.1	intergenic
AGAGGAGCGCCCGGCCCCCGCGG	3	0.002831079	NC_013896.1	intron

* Off-target scores are calculated by CRISPOR (Doench JG. *et al*., 2016), and the scores greater than 0.2 are green-highlighted.
Blue letters of sgRNA sequence are PAM, and red letters indicate mismatch residue.
